# Supplementary material for: Mammalian cell growth characterisation by a non-invasive plate reader assay
Source: Nat Commun. 2024 Jan 2;15:57. doi: 10.1038/s41467-023-44396-4 (PMC10761699; doi:10.1038/s41467-023-44396-4)
Supplement: Supplementary file 1 — Supplementary information [file 41467_2023_44396_MOESM1_ESM.pdf]

Supplementary Information

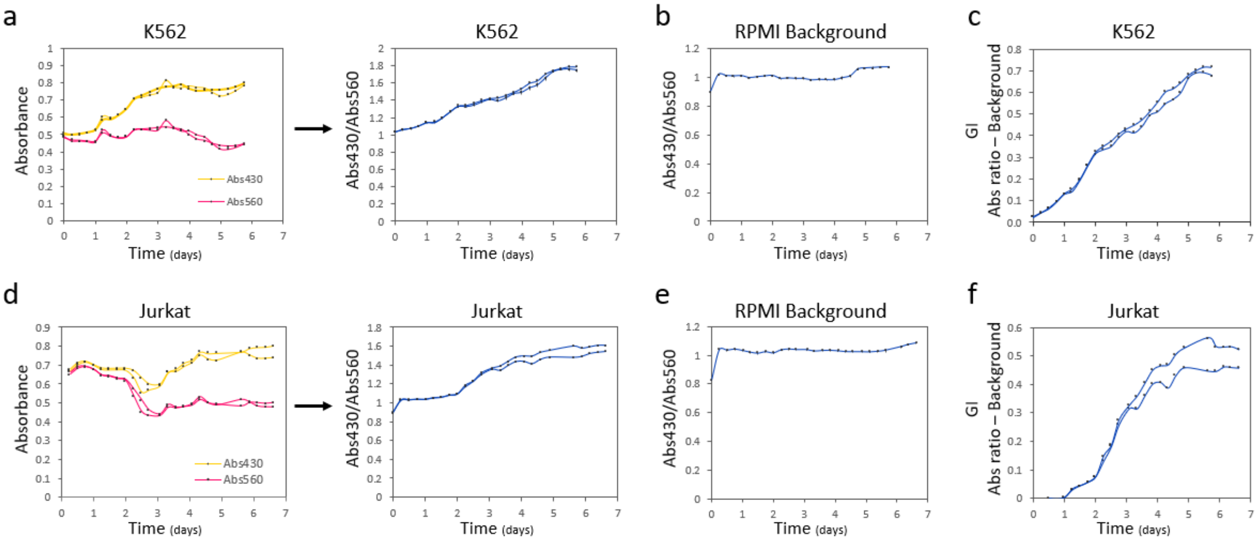

Figure S1. GI calculation for suspension cells from phenol red individual plate reader absorbance values. a) Representative duplicate variations of Abs_{430} (yellow) and Abs_{560} (pink) over time (left) for RPMI medium when K562 cells are cultured. Resulting Abs_{430} over Abs_{560} ratios (right, blue) over time. **b)** Average background and error of the mean for Abs_{430}/Abs_{560} ratio of RPMI over time (n=8) in control wells containing no cells. **c)** Representative duplicate GI profiles over time for K562 cells, resulting from Abs_{430}/Abs_{560} ratio normalised to RPMI background. **d)** Representative duplicates of Abs_{430} (yellow) and Abs_{560} (pink) over time (left) for RPMI medium over cultured Jurkat cells. Resulting Abs_{430} over Abs_{560} ratios (right, blue) over time. **e)** Background and error of the mean for Abs_{430}/Abs_{560} ratio of RPMI over time (n=8) in wells containing no cells. **f)** Representative duplicate GI profiles over time for Jurkat cells, resulting from Abs_{430}/Abs_{560} ratio normalised to RPMI background. Source data are provided as a Source Data file.

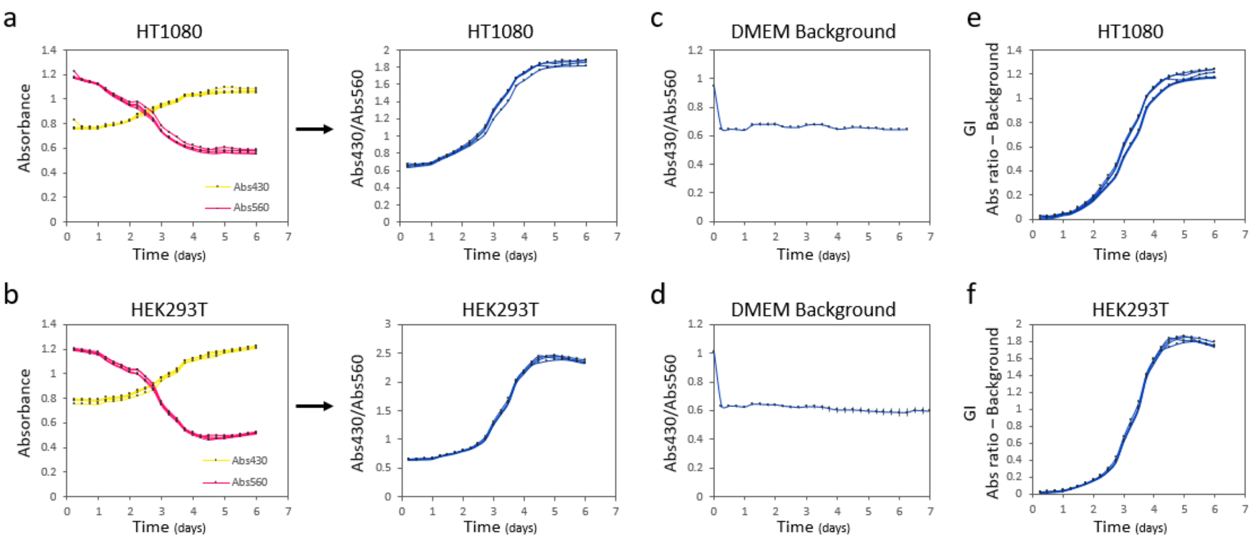

Figure S2. GI calculation for adhesion cells from phenol red individual plate reader absorbance values. Abs_{430} (yellow) and Abs_{560} (pink) over time (left) for DMEM medium when HT1080 **(a)** and HEK293T **(b)** cells are cultured. Resulting Abs_{430} over Abs_{560} ratio (right, blue) over time (n=4). Average background and error of the mean for Abs_{430}/Abs_{560} ratio of DMEM over time (n=3) in control wells containing no cells **(c**, **d)** GI profiles over time for HT1080 **(e)** and HEK293T **(f)** cells, resulting from Abs_{430}/Abs_{560} ratio normalised to DMEM background (n=4). Source data are provided as a Source Data file.

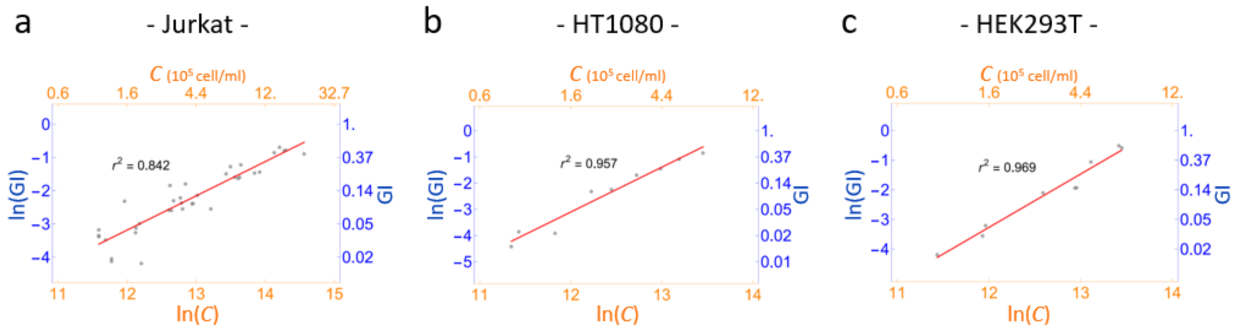

Figure S3. Linear relation of ln(GI) and ln(*C*) for Jurkat, HT1080 and HEK293T cells grown at 37°C with Glu. Graphs representing the relation between GI and *C* for **a)** Jurkat, **b)** HT1080 and **c)** HEK293T cells in logarithmic scale. The red solid line is the best fit of the data in linear region for which r^2 values are indicated. Linear fit equations are reported in Table S2. Numbers of biological repeats for each sample are reported in Table S3. Data analysis is described in the Methods section and in Supplementary Note 1. Source data are provided as a Source Data file.

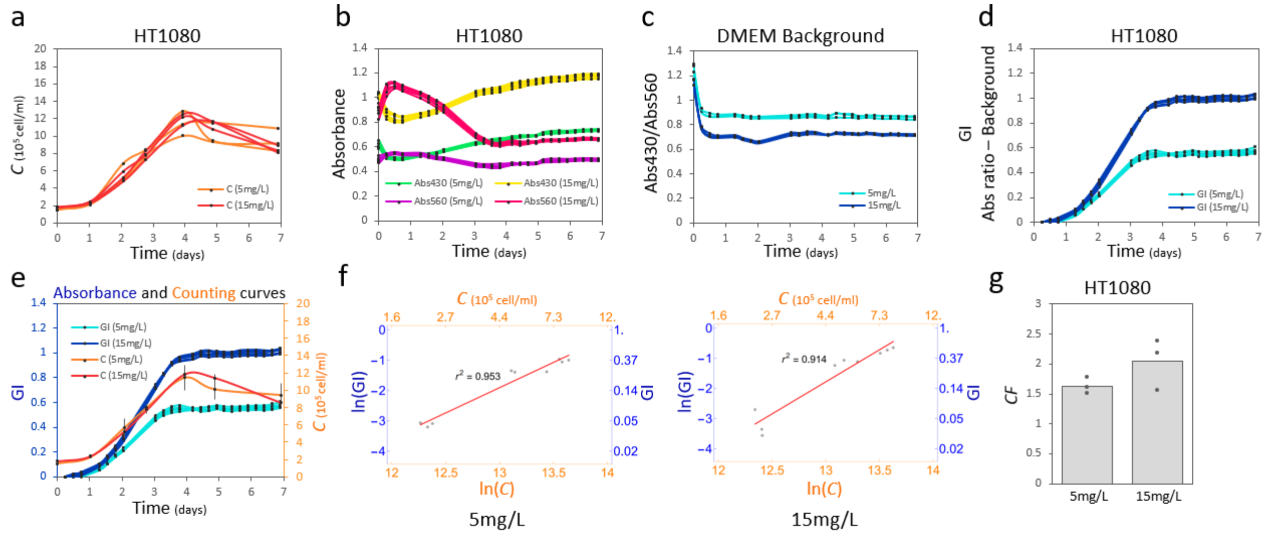

Figure S4. Effect of phenol red concentration on GI curves for HT1080 cells. a) Individual growth curves resulting from counting HT1080 cells cultured with 5mg/L (light orange) or 15mg/L (dark orange) phenol red (n=3). **b)** Overtime measure of Abs_{430} (green and yellow lines) and Abs_{560} (purple and pink lines) for HT1080 cells cultured in DMEM containing 5mg/L or 15mg/L phenol red (n=5). **c)** Overtime Abs_{430}/Abs_{560} ratio for DMEM background in control wells containing no cells in the presence of 5mg/L (light blue) or 15mg/L (dark blue) phenol red (n=3). **d)** GI profiles over time for HT1080 cells cultured in DMEM containing 5mg/L (light blue) or 15mg/L (dark blue) of phenol red (n=5). **e)** Representative growth curves resulting from phenol red acidification (GI, blue, left vertical axis) and averaged cell counts (*C*, orange, right vertical axis) of HT1080 cells cultured with 5mg/L (light blue and light orange) or 15mg/L (dark blue and dark orange) of phenol red (n=3). Numbers of biological repeats for each sample are reported in Table S3. For cell counts, data are presented as mean values +/- SD. **f)** Linear relation between ln(GI) and ln(*C*) for HT1080 cells when cultured in DMEM containing 5mg/L (left) or 15mg/L (right). The red line is the best fit of data within the linear region. Coefficient of determinations are indicated (r^2=0.914 for 5mg/L and r^2=0.953 for 15mg/L). **e)** Bar plot of CF for HT1080 cells cultured in DMEM containing either 5mg/L or 15mg/L phenol red. The height of the bar represents the mean value of the single replicates shown as black dots. All replicates can be found in Supplementary data file 1 while growth rate and CF are reported in Supplementary data file 2. Source data are provided as a Source Data file.

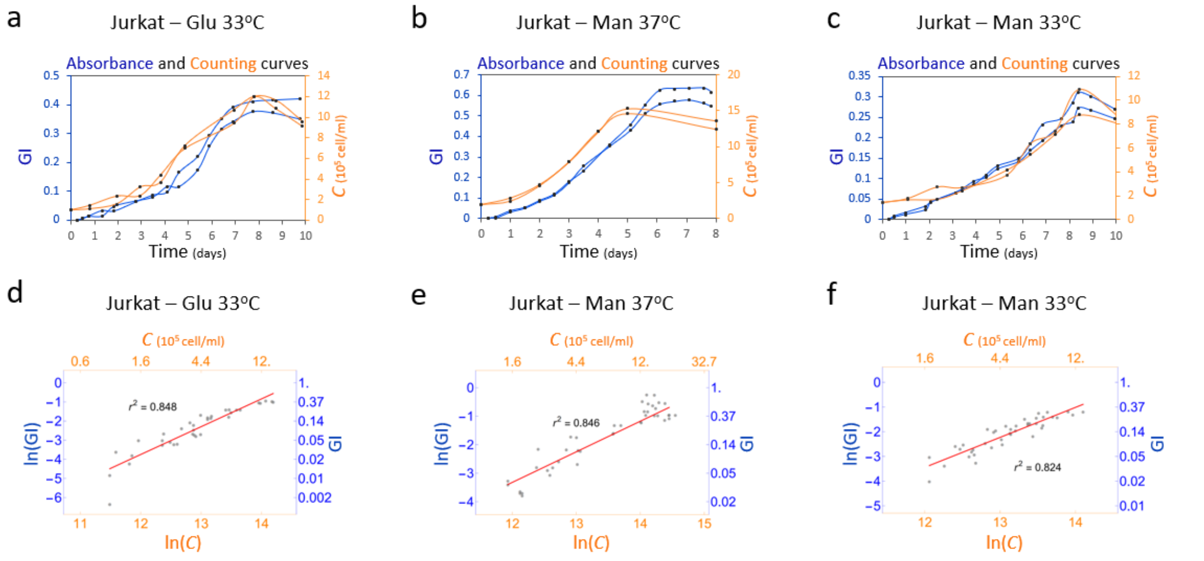

Figure S5. Growth of Jurkat suspension cells under different conditions can be characterised by a plate reader assay. Representative GI (blue, left vertical axis) and *C* (orange, right vertical axis) profiles over time for Jurkat cells grown with glucose (Glu) at 33ºC **(a)** or mannose (Man) at either 37ºC **(b)** or 33ºC **(c)**. All biological replicates can be found in Supplementary data file 1. Relation between GI and C for Jurkat cells grown in presence of Glu at 33ºC **(d)** or Man at either 37ºC **(e)** or 33ºC **(f)** in logarithmic scale. The red solid line is the linear fit of the data in the linear region for which r^2 values are indicated. Linear fit equations are reported in Table S2. Numbers of biological repeats for each sample are reported in Table S3. Data analysis is described in the Methods section and in Supplementary Note 1. Source data are provided as a Source Data file.

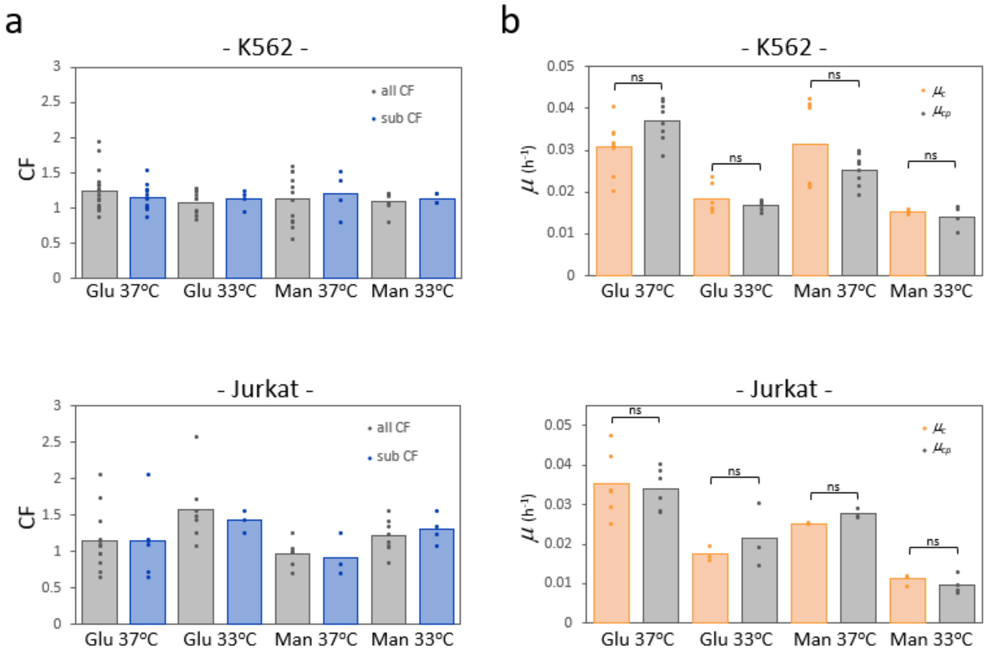

Figure S6. Reproducibility test of plate reader growth characterisation for K562 and Jurkat cells grown in different conditions. To test the automatable capability of our plate reader assay, the data set obtained for each growing condition was split. CF from one subset were averaged together (Table S5) to convert μ_p into μ_{cp} in the remaining data subset. **a)** Bar plots of all (grey) or a subset (sub, blue) of CF for K562 (top) and Jurkat (bottom) cells grown in presence of either glucose (Glu) or mannose (Man) at 37ºC or 33ºC. **b)** Bar plots of μ_c (orange) and μ_{cp} (grey), calculated as μ_p x CF average, for K562 (top) and Jurkat (bottom) cells grown in presence of either Glu or Man at 37ºC or 33ºC are shown. A two-sided t-test indicated that average CF of all or one subset are compatible ($p>0.05$) and that μ_{cp} are compatible with actual μ_c (ns, non-significant: $p>0.05$). The height of the bars represents the average value of the single replicates shown as dots. Numbers of biological repeats for each sample are reported in Table S3. Data analysis is described in the methods section and in Supplementary Note 1. Exact p values and source data are provided as a Source Data file.

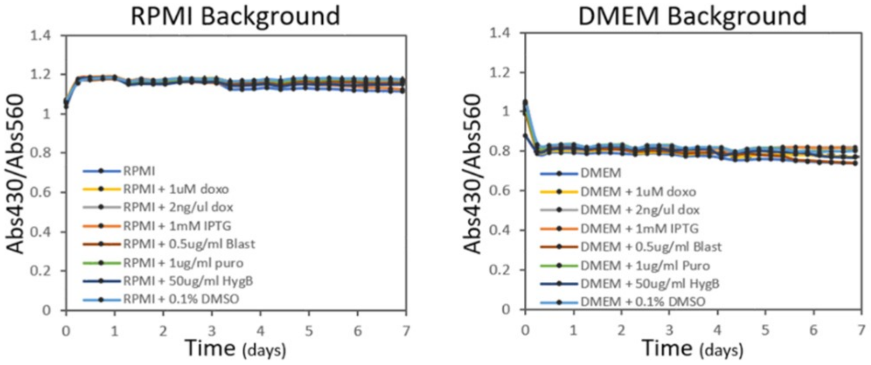

Figure S7. Compounds effect on media acidification. Average Abs_{430}/Abs_{560} ratios and +/- SEM over time of RPMI (left) and DMEM (right) medium supplemented with either 1uM doxorubicin (doxo), 2ng/µl doxycycline (dox), 1mM IPTG, 0.5ug/ml blasticidin (Blast), 1ug/ml puromycin (Puro), 50ug/ml hygromycin B (HygB) or 0.1% DMSO at T0 (n=4). Source data are provided as a Source Data file.

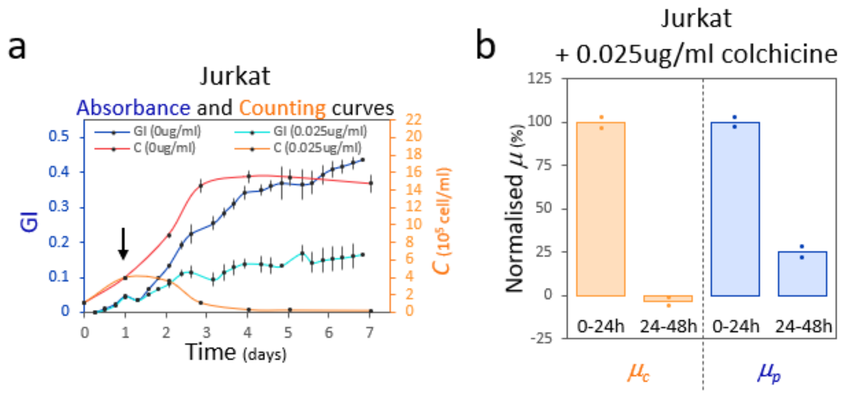

Figure S8. Jurkat cells treated with colchicine. Analysis of growth rate changes in Jurkat cells untreated (0ug/ml) or treated (0.025ug/ml) with colchicine added at 24h after the start of the assay (black arrow). **a)** Growth curves resulting from phenol red acidification (GI, blue, left vertical axis) and cell counts (*C*, orange, right vertical axis) of Jurkat cells cultured with colchicine 0ug/ml (dark blue and dark orange) or 0.025ug/ml (light blue and light orange) (n=4). Data are presented as mean values +/- SEM. **b)** Histograms of relative μ_c (orange, left) and μ_p (blue, right) values of Jurkat treated cells. Data show μ_c and μ_p for the 0-24 hours time window (preceding colchicine addition and for the 24-48 hours time window (after colchicine addition). Data were normalised to 100% growth rate in the 0-24 hours window. The height of the bar represents the mean value of the single replicates shown as dots. After colchicine addition, μ_c is decreased by ~100%, and μ_p is decreased by ~75%. -Growth rates are reported in Supplementary data file 2 while all replicates can be found in Supplementary data file 1. Source data are provided as a Source Data file.

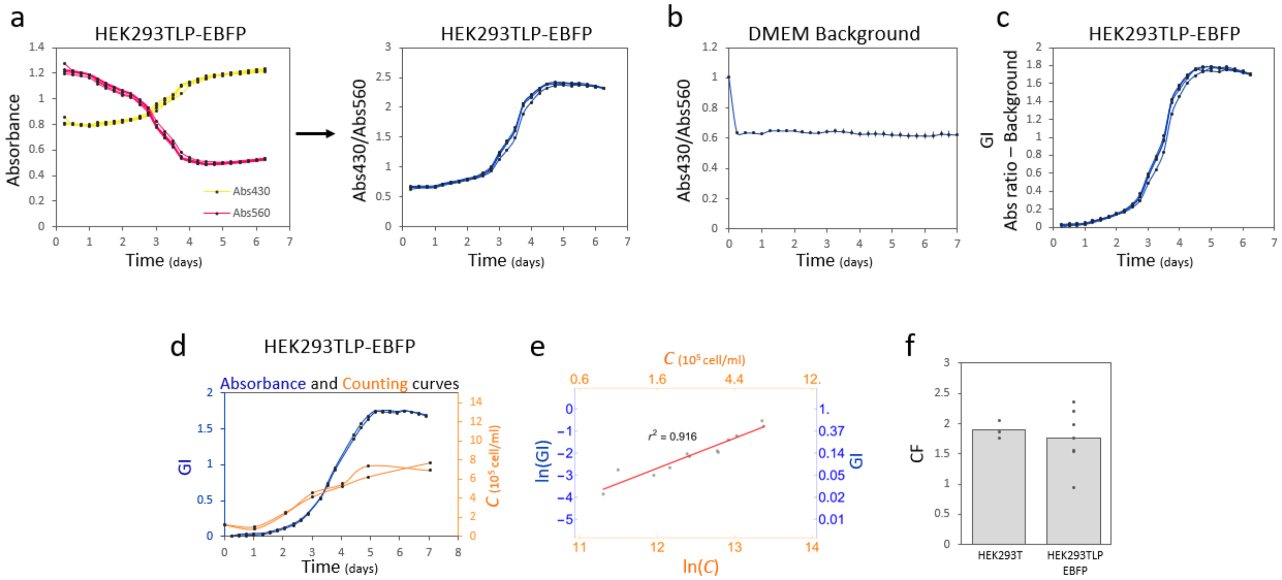

Figure S9. Growth of HEK293TLP-EBFP cells can be characterised with the plate reader-based assay. a) Abs_{430} (yellow) and Abs_{560} (pink) over time (left) for DMEM medium when HEK293TLP-EBFP cells are cultured. Resulting Abs_{430} over Abs_{560} ratio (right, blue) over time (n=4). **b)** Average background and error of the mean for Abs_{430}/Abs_{560} ratio of DMEM over time (n=3) in control wells containing no cells. **c)** GI profiles over time for HEK293TLP-EBFP cells, resulting from Abs_{430}/Abs_{560} ratio normalised to DMEM background (n=4). **d)** Representative growth curves resulting from phenol red acidification (GI, blue) and cell counts (*C*, orange). All biological replicates can be found in Supplementary data file 1. **e)** Relation between GI and *C* in logarithmic scale. Red solid line is the linear fit of the data in linear region for which r^2=0.916. The linear fit equations are reported in Table S2. **f)** Bar plot of CF for HEK293TLP-EBFP cells compared to their HEK293T parental cell line. The height of the bar represents the average value of the single replicates shown as black dots. Numbers of biological repeats for each sample are reported in Table S3. Data analysis is described in the Methods section and in Supplementary Note 1. Source data are provided as a Source Data file.

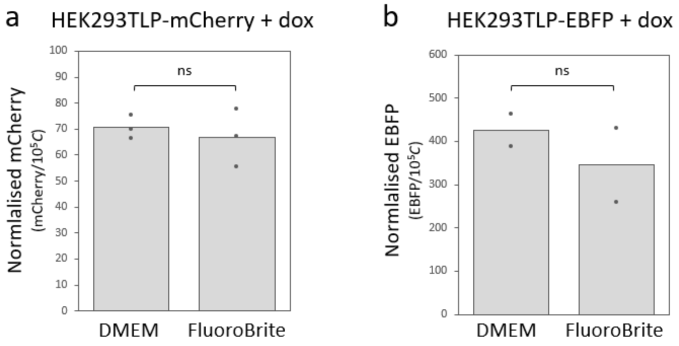

Figure S10. Fluorescence readout for mCherry and EBFP in presence and absence of phenol red in the medium. Cells were grown in the incubator with and without induction with 2ng/µl dox for 2 days **(a)** (mCherry, n=3) and 3 days **(b)** (EBFP, n=2). Cells were then resuspended in medium with (DMEM) and without (FluoroBrite) phenol red and fluorescence was measured at the plate reader. Cell counts were performed in parallel. A two-sided t-test indicated that average fluorescence level in DMEM and FluoroBrite are compatible (ns, non-significant: p>0.05). Data and statistical analysis are described in the Methods section. Raw values are reported in the source data file. Source data are provided as a Source Data file.

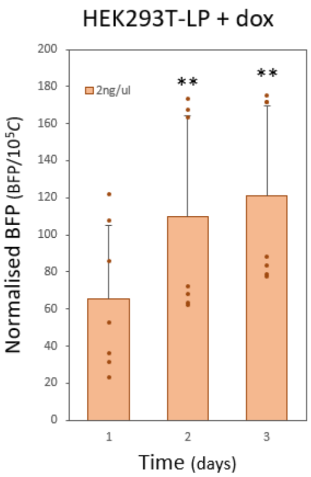

Figure S11. EBFP expression level in HEK293TLP-EBFP cells after dox induction. Bar plot of normalised fluorescence per cell (calculated as EBFP/10^5C) for HEK293TLP-EBFP cells induced with 2ng/µl dox over three days. Data are presented as mean values + SD with single replicates shown as dots. A two-sided t-test indicated that normalised EBFP levels increase significantly each day (**: $p<0.01$). Numbers of biological repeats for each sample are reported in Table S3. Data and statistical analysis are described in the Methods section. Exact p values and source data are provided as a Source Data file.

Supplementary tables

Cell line and growing condition	GI interval of linearity [GI_{min}; GI_{max}]	*C* interval of linearity [C_{min}; C_{max}] (10^5 cell/ml)
K562 Glu37°C	[0.034; 0.42]	[1.05; 10.9]
Jurkat Glu 37°C	[0.015; 0.51]	[1.08, 21.1]
HT1080 Glu 37°C	[0.012; 0.43]	[0.84; 6.95]
HEK293T Glu 37°C	[0.015; 0.61]	[0.93; 6.93]
HEK293TLP-EBFP Glu 37°C	[0.021; 0.59]	[0.81; 6.45]
K562 Glu 33°C	[0.022; 0.40]	[1.08; 10.3]
Jurkat Glu 33°C	[0.0018,0.39]	[0.975; 14.6]
K562 Man 37°C	[0.027; 0.24]	[0.92; 9.7]
Jurkat Man 37°C	[0.05; 0.43]	[2.38; 18.9]
K562 Man 33°C	[0.019; 0.24]	[1.08; 9.37]
Jurkat Man 33°C	[0.018; 0.305]	[1.73; 13.3]

TABLE S1. Intervals of linearity for each condition: region of fastest growth for *C* growth curves and corresponding values for GI growth curves.

Cell line and growing condition	Fit ln(GI) = *m**ln(*C*) + *q*	r^2	Fit parameter (*q* ± err*q*)	Fit parameter (*m* ± err*m*)
K562 Glu 37°C	ln(GI) = 0.99*ln(C) - 14.6	0.893	(-14.6 ± 0.6)	(0.99 ± 0.04)
Jurkat Glu 37°C	ln(GI) = 1.03*ln(C) - 16	0.842	(-16 ± 1)	(1.03 ± 0.08)
HT1080 Glu 37°C	ln(GI) = 1.71*ln(C) - 23.6	0.957	(-23.6 ± 1.7)	(1.71 ± 0.14)
HEK293T Glu 37°C	ln(GI) = 1.80*ln(C) - 24.9	0.969	(-24.9 ± 1.5)	(1.80 ± 0.12)
HEK293TLP-EBFP Glu 37°C	ln(GI) = 1.36*ln(C) - 19.1	0.916	(-19.1 ± 1.6)	(1.36 ± 0.13)
K562 Glu 33°C	ln(GI) = 0.98*ln(C) - 14.7	0.752	(-14.7 ± 1.0)	(0.98 ± 0.08)
Jurkat Glu 33°C	ln(GI) = 1.45*ln(C) - 21.2	0.848	(-21.2 ± 1.4)	(1.45 ± 0.11)
K562 Man 37°C	ln(GI) = 0.87*ln(C) - 13.4	0.855	(-13.4 ± 0.8)	(0.87 ± 0.06)
Jurkat Man 37°C	ln(GI) = 1.07*ln(C) - 16.2	0.846	(-16.2 ± 1.3)	(1.07 ± 0.10)
K562 Man 33°C	ln(GI) = 1.01*ln(C) - 15.5	0.887	(-15.5 ± 0.7)	(1.01 ± 0.06)
Jurkat Man 33°C	ln(GI) = 1.21*ln(C) - 18.0	0.824	(-18.0 ± 1.2)	(1.21 ± 0.09)

TABLE S2. Results of the best fits for determining the relation between ln(GI) and ln(*C*) for all growing conditions with corresponding r^2 and mean parameter values +/- SEM.

	Number of biological repeats	
**Cell line and growing condition**	Plate reader	Cell counting
K562 Glu 37°C	20	20
Jurkat Glu 37°C	12	12
HT1080 Glu 37°C	3	3
HT1080 (15mg/L phenol red) Glu 37°C	3	3
HT1080 (5mg/L phenol red) Glu 37°C	3	3
HEK293T Glu 37°C	3	3
HEK293TLP-BFP Glu 37°C	7	7
K562 Glu 33°C	10	10
Jurkat Glu 33°C	7	7
K562 Man 37°C	12	12
Jurkat Man 37°C	6	6
K562 Man 33°C	8	8
Jurkat Man 33°C	8	8
HT1080 + doxo (0, 10, 25, 50, 75, 100, 500, 750 and 1000nM)	2-4 / [doxo]	2-4 / [doxo]
K562 + doxo (0, 10, 25, 50, 75, 100, 500, 750 and 1000nM)	4 / [doxo]	4 / [doxo]
HEK293T-LP + dox (0, 0.1, 0.25, 0.5, 1 and 2ng/µl)	7 / [dox]	-

Tables S3. Number of biological repeats per experiments with plate reader and cell counting protocol summary.

	Average ± err
K562 Glu 37°C	(1.24 ± 0.06)
Jurkat Glu 37°C	(1.15 ± 0.12)
HT1080 Glu 37°C	(1.76 ± 0.10)
HEK293T Glu 37°C	(1.89 ± 0.08)
HEK293T-LP Glu 37°C	(1.45 ± 0.18)

Tables S4. Average CF +/- SEM for cells grown at 37°C with Glu.

	Average of all CF	Average of CF subset
K562 Glu 37°C	(1.24 ± 0.06)	(1.16 ± 0.05)
K562 Glu 33°C	(1.09 ± 0.05)	(1.12 ± 0.06)
K562 Man 37°C	(1.13 ± 0.10)	(1.12 ± 0.16)
K562 Man 33°C	(1.10 ± 0.05)	(1.14 ± 0.04)
Jurkat Glu 37°C	(1.15 ± 0.12)	(1.14 ± 0.20)
Jurkat Glu 33°C	(1.58 ± 0.18)	(1.49 ± 0.06)
Jurkat Man 37°C	(0.96 ± 0.08)	(0.92 ± 0.17)
Jurkat Man 33°C	(1.21 ± 0.08)	(1.30 ± 0.10)

Tables S5. Average CF values +/- SEM across the different growing conditions of K562 and Jurkat cells.

Supplementary Note 1. Computational pipeline for automated data analysis

<u>**Content:**</u>

- **Computational pipeline for automated data analysis: Growth rates, conversion factors and relation between ln(GI) and ln(*C*).**
- **Pseudo-codes of algorithms developed for data analysis.**
- **On the relation between the linear fit of ln(GI) vs ln(*C*) and CF.**
- **Analysis of cells treated with doxo.**

Computational pipeline for automated data analysis: Growth rates, CF and relation between ln(GI) and ln(*C*).

The procedure that we adopted to obtain quantitative information relating plate reader and manual counts measurements of cell populations' growth originate from the following observation. Let's consider K562 growth curves measured both with manual counts (Fig. S12a) and the plate reader (Fig. S12b) expressed in logarithmic scale and binned every 50 hourslin (each colour of Fig. S12a-b represents a different temporal range). As time increases, first both ln(*C*) and ln(GI) increase their values covering almost all their range of variability in ~50 hours (this is the exponential phase of growth for both *C* and GI, red dots in Fig. S12a-c). Then, *C* saturates and decreases while GI only saturates (orange, green and blue dots). Therefore, as depicted in Fig. S12c, there is a temporal order in the way the data dispose in the plane ln(GI) vs ln(*C*): a first linear increase is then followed by a "simil-hysteresis" trend due to the different behaviour of the two quantities in the late growth phase. Based on this observation, we developed a method for quantifying i) the linear relation between ln(GI) and ln(*C*) that allows to convert measured GIs into cell concentrations, *C* and ii) the conversion factor (CF) that allows to convert growth rate measured in plate reader's growth curve (μ_p) into the growth rate obtained when cells are manually counted (μ_c).

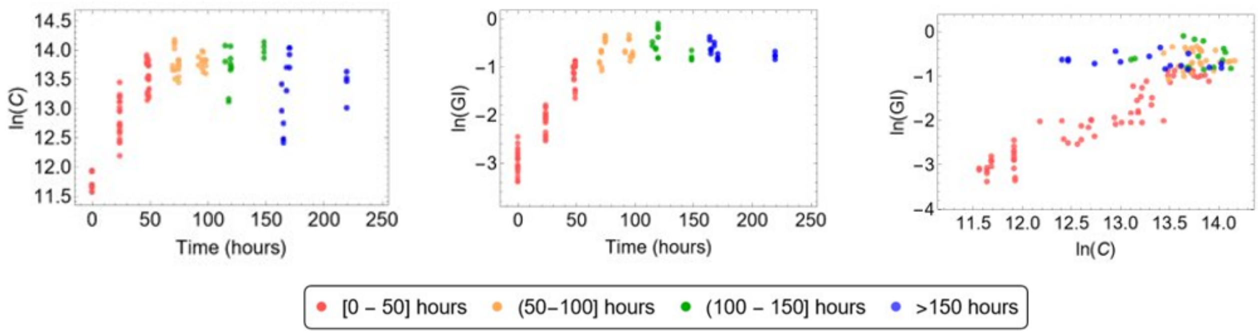

Figure S12. Measuring growth curves with manual counts and plate reader. Growth of K562 cells in Glucose at 37°C measured during time with **a)** manual counts and **b)** plate reader shown in logarithmic scale. Data are binned in time-windows of 50 hours, each colour represents one time-window. **c)** y-axis of panel b) (ln(GI)) as a function of the y-axis of panel a) (ln(*C*)). The data in the first 50 hours (in red) cover the region of fastest growth of curves in panel a) and correspond to the data shown in Figure 1d. Source data are provided as a Source Data file.

Our method is based on the three algorithms briefly described below (pseudo-codes in the following Tables I-III).

ALGORITHM 1 allows to select the time window corresponding to the fastest growth in manual counts growth curves and to compute the growth rate in such time-window. To do this, the algorithm: i) first performs linear fits of at least 3 consecutive points in sliding windows that start from time 0 and slide until the end of the growth curve (lines 1 – 10 in Table I). ii) Then, the algorithm selects the range of data points whose linear fit gives the maximum slope (line 12). In this way, the lower, the upper data limits, and the number of points to fit are optimized in the regression. The slope of the linear fit of these data points is the growth rate of the exponential phase (μ_c). This procedure is performed on curves obtained with manual counts and expressed in log scale, normalized to their initial value. The choice of having at least 3 data points has been based on the interest in estimating the average growth rate in the exponential phase and not the maximum growth rate. The latter, given the resolution of our manual counts, could be more easily biased by eventual experimental variability in cell counts. However, the supplied script for data analysis allows the user to choose the minimum number of data points on which to perform the fit.

In our analysis, such number (i.e., the parameter ***minNdata*** in Table I-III) has been set to 3 for all the growth conditions at 37°C, except for i) Jurkat Man 37°C where it was set to 4 and ii) Jurkat and K562 growing at 33°C where it was set to 5. The choice of the two values at points i) and ii) was driven by a longer duration of the exponential phase in the analysed conditions with respect to the other conditions.

Concerning data treated with doxo, the described procedure was applied to the data treated with all concentrations of doxo (by starting with at least 2, 3 or 4 data points, depending on the condition) except for HT1080 + doxo 1 μM. In this latter case, since the cells were almost not-growing, the automatic procedure was biased by fluctuations in cell counts. For this reason, we manually estimated the time window for calculating the growth rates as including the first 3 data points of each curve.

ALGORITHM 2 aims at computing the CF. For each growth curve measured with both *C* and GI, it relies on the outputs of *ALGORITHM 1* to i) first selecting the time points in GI growth curve that correspond to the fastest growth in *C* growth curve (lines 1,2 in Table II), then ii) it computes the growth rate in GI growth curve (μ_p) as the slope of the linear fit among such points (line 3), and finally iii) calculates the conversion factor CF = μ_p/μ_c (lines 4-5).

ALGORITHM 3 quantifies the linear relation between ln(GI) and ln(*C*) in their region of fastest growth. To do this, as *ALGORITHM 2* it i) first uses the output of *ALGORITHM 1* to identify the values of GI corresponding to the region of fastest growth in *C* growth curve (lines 1-6 in Table III) and then ii) computes the linear fit between ln(GI) and ln(*C*) (lines 7,8).

Pseudo-codes of algorithms developed for data analysis.

ALGORITHM 1. SELECTION OF GROWTH CURVE TIME POINTS CORRESPONDING TO THE FASTEST GROWTH

Inputs:

1. Normalized logarithmic growth curves C vs time, ***m*** time-points: ***dataC*** = (timeC, ln(C/C0))
2. ***minNdata*** = minimum number of data to fit.

Outputs:

1. ***idxμC*** = positions of time-points with the maximum slope
2. ***μC*** = value of the maximum slope
3. ***bestFit*** = best linear fit giving the maximum slope

Initialization of variables: assign 0 to variable ***i***

Initialization of matrices:

slopes = matrix of size (***ndata***) x (***ndata*** - ***ndataToadd***);

R2s = matrix of size (***ndata***) x (***ndata*** - ***ndataToadd***);

indexesfits = matrix of size (***ndata***) x (***ndata*** - ***ndataToadd***); # this will store the position of the first and the last data in the growth curve used for each fit.

```
ndataToadd = minNdata – 1;
while (i ≤ m) do
    for (j = ndataToadd to (m – i))
        indexesfits = (i, i + ndataToadd);
        select dataToFit = dataC at positions from i to (i + ndataToadd)
        linear fit of dataToFit;
        save the slope of the linear fit in the matrix slopes;
        save the R2 of the linear fit in the matrix R2s;
    end
end
μC = max(slopes);
IdxμC = find the indices in indexesfits corresponding to the fit with the maximum slope
return: IdxμC, μC
```

Table I: Pseudo-code for selecting the timepoints corresponding to the region of fastest growth in manual counts (*C*) growth curves and computing μ_c.

ALGORITHM 2. COMPUTE THE CONVERSION FACTOR	
Inputs:	***1.*** First need to run ALGORITHM 1. ***Inputs for ALGORITHM 1:***<br>***dataC*** = (timeC, ln(C/C0))<br>***minNdata*** = minimum number of data to fit<br>2. ***datalnGI*** = (timeGI, ln(GI/GI0))<br>Note: For GI only time points corresponding to C must be considered.
Outputs:	***μC***, ***μP, CF***
1	Run ***ALGORITHM 1*** with ***dataC*** and ***minNdata*** as inputs.
2	Use the output ***IdxμC*** of ***ALGORITHM 1*** to select the time points of the growth curves GI corresponding to the maximum slope in C:<br>***datalnGImaxSlope*** = datalnGI(***IdxμC***);
3	linearFit of ***datalnGImaxSlope***;
4	***μP*** = slope of the linear fit;
5	***CF*** = ***μP***/***μC***;

Table II: Steps for computing μ_p and the conversion factor (CF).

ALGORITHM 3. LINEAR RELATION BETWEEEN ln(GI) and ln(C)	
Inputs:	***1.*** First need to run ***ALGORITHM 1***. ***Inputs for ALGORITHM 1:***<br>***dataC*** = (timeC, ln(C/C0))<br>***minNdata*** = minimum number of data to fit<br>2. ***dataC*** = (timeC, C)<br>3. ***dataGI*** = (timeGI, GI)<br>Note: For GI only time points corresponding to C must be considered.
Outputs:	***Linear fit ln(GI) vs ln(C), its r^2 and edges of the linear region (C_{min}, C_{max}, GI_{min}, GI_{max})***
1	Run ***ALGORITHM 1*** with ***dataC*** and ***minNdata*** as inputs.
2	Use the output ***IdxμC*** of ***ALGORITHM 1*** to select the C and GI values corresponding to the maximum slope in C:<br>***CmaxSlope*** = dataC(***IdxμC***);<br>***GImaxSlope*** = dataGI(***IdxμC***);
3	C_{min} = min(***CmaxSlope***); # lower bound for C in the region of linearity
4	C_{max} = max(***CmaxSlope***); # upper bound for C in the region of linearity
5	GI_{min} = min(***GImaxSlope***); # lower bound for GI in the region of linearity
6	GI_{max} = max(***GImaxSlope***); # upper bound for C in the region of linearity
7	LinearFit of ***datalnGIvsC*** = (ln(***CmaxSlope***), ln(***GImaxSlope***))
8	***r^2*** = r^2 of the linear fit;

Table III: Steps for computing the linear relation between ln(GI) vs ln(*C*), its r^2, the boundaries of linearity.

On the relation between the linear fit of ln(GI) vs ln(C) and CF.

Since the linear relation between ln(GI) and ln(C) holds within the exponential phase, its slope can be directly related to CF. Indeed, according to our analysis, in the exponential phase of growth, the following expressions hold:

1. $\ln(C/C0) = a + \mu_C * t \rightarrow \ln(C) = a + \ln(C0) + \mu_C * t = a' + \mu_C * t$
2. $\ln(GI/GI0) = b + \mu_P * t \rightarrow \ln(GI) = b + \ln(GI0) + \mu_P * t = b' + \mu_P * t$
3. $\ln(\mathrm{GI}) = m * \ln(C) + q$

From (1), $t = (\ln(C) - a')/\mu_C$ that substituted in (2) gives :

4. $\ln(\mathrm{GI}) = (\mu_P/\mu_C)*\ln(C) + (b' - a' * \mu_P/\mu_C)$

From (3) and (4) it follows $m = \mu_P/\mu_C$ that corresponds to our definition of CF.
The compatibility between m and CF is shown in Figure S13a-c where, per each experiment discussed in Figure 1 and 2, points having as coordinates CF and m +/- its error obtained with the linear fit ln(GI) vs ln(C) lie on the bisector.

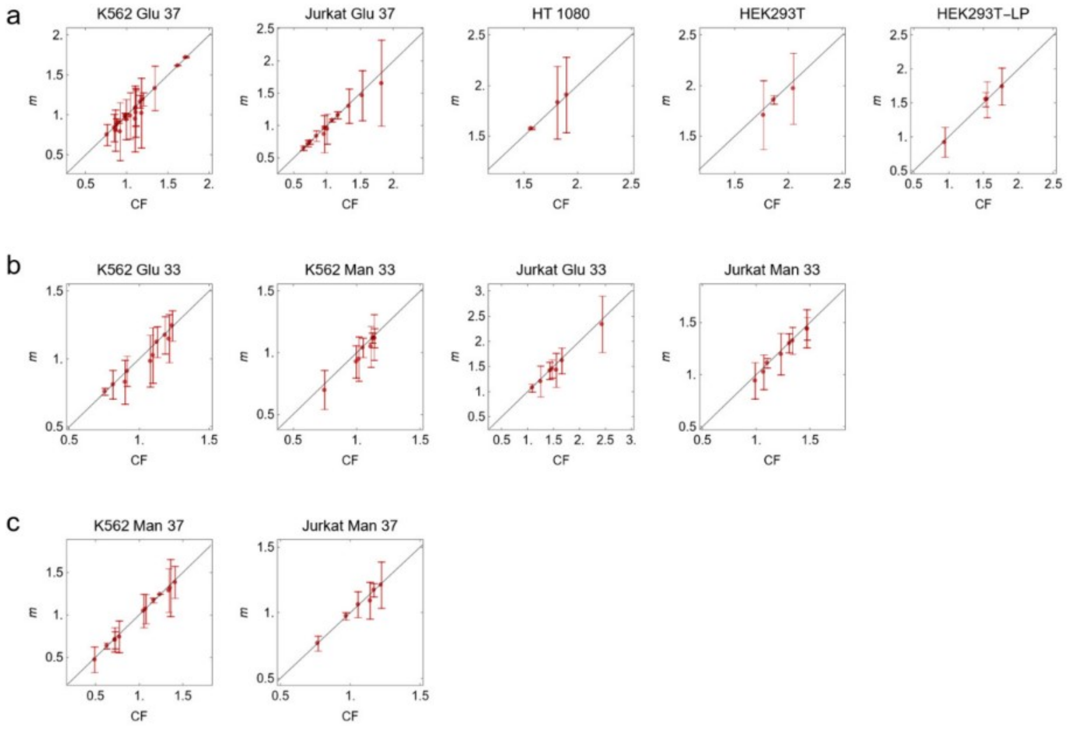

Figure S13. Relation between CF and *m* per each individual growth curve. The parameter m (+/- SEM) obtained from the linear fit of ln(GI) vs ln(C) and CF = μ_p/μ_c for each individual growth curve in red. In black is the bisector (i.e., m = CF). In all cases the bisector passes within the error bars. In **(a)** are the conditions analysed in Figure 1 of the main text together with HEK293T-LP; in **(b)** are the growth conditions at 33°C, in **(c)** K562 and Jurkat growing in Mannose (Man) at 37°C. Source data are provided as a Source Data file.

Thanks to this, CF found during the calibration of the studied cell line, can be used for calculating the growth rate within the exponential phase.

From (3) it follows that GI = $\exp(q)*C^m$ --> $C = (GI/\exp(q))$^$(1/m)$.

Analysis of cell lines treated with doxo.
To obtain the values shown in Figure 3c and 3d, we proceeded with the following three steps. First, we computed the growth rates in exponential phase with the analytic procedure we previously adopted in *Enrico Bena et al* [1]. Briefly, the growth curves measured with time resolution of 6 hours were fitted with a modified logistic function which parameters were the lag time, the exponential maximum growth rate and the level of saturation. Then, the edges of the exponential phase of growth were analytically determined as the intersection between the tangent to the fitted curve at the inflection point and the x-axis, as lower bound, and the horizontal line at the saturation level, as the upper bound. Then, the growth rate within the exponential phase was determined as the linear fit of the data within such phase.
Secondly, per each run, we computed the average growth rate of cells grown with 0 nM of doxo and thirdly, we normalized all the growth rates to the average value of the first run (Exp1) and expressed it in percentage.

Supplementary Note 2
Variability observed in CF and *m*

The calculated values of CF and m in the present work were characterised by some variability. To explain this, we would like to point the readers to our previously published work (*Enrico Bena et al. 2021*[1]), in particular Figure 3c where we considered the dependence of mammalian cells growth rate in exponential phase on the initial culture seeding concentration.

It was found that there exist a range of seedings, around to 10^5 cells/ml (which is exactly the recommended seeding for our cell lines and the one used for experiments presented in our manuscript), for which the variability in growth rates within the exponential phase (lambda_max) is maximum (in the figure each dot represents the maximum growth rate of a population of Jurkat cells and the same holds for K562 cells). This means that growth curves generated with that initial seeding will be highly variable in terms of maximal growth rate (much more than curves generated with lower seeding N_0, such as 10^3 cells/ml). If one used a few of these curves to generate the model and obtain CF, the variability in the growth rates will reflect on the amplitude of the error bar over the CF. Thus, experiments performed with initial seeding different by orders of magnitude must be considered as performed in different growth conditions, requiring initial characterisation as explained in the main manuscript.

From the above, we consider that for the seeding more widely adopted in lab-based experiments and advised by manufacturer`s instructions (~10^5 cells/ml) there exist an intrinsic variability. In our work we show how, besides this given intrinsic variability, we are able to measure CF, providing an easy and quick method for mammalian cell growth tracking that is less time consuming and provides higher throughput than traditional cell counting. The price to be paid is a confidence interval higher than that we would have in the case of lower seeding. On the contrary, for lower-seeding experiments, the price to be paid would be a much longer and variable lag time, leading to single growth experiments taking up to 20-30 days, as we previously showed. Please refer to Figure 2a in *Enrico Bena et al*[1] to see the plot displaying variability in the lag time, and thus in the overall experiment timeline, as a function of N_0.

Supplementary Note 3

Accuracy in converting GI into *C* compared to the accuracy in converting bacteria's OD into *C*.

To quantify the accuracy of our method in estimating cell concentration from absorbance measurements, we compared it to the accuracy in estimating bacteria concentration from OD_{600} measurements. In order to do so, we quantified the accuracy in cell concentration estimates as defined in *Beal et al*[2] and compared our results to those obtained there for bacteria. In *Beal et a*l, the authors defined the accuracy as the ratio between the estimated mean fluorescence per cell (fluorescence from *E. coli* normalised by calibrated OD measurements) and the mean fluorescence per cell measured by calibrated flow cytometry. We reasoned that, in our case, this would mean to compare the mean cell count predicted by our calibration method (C_p or $C_{predicted}$) to the mean cell count effectively measured (C or $C_{measured}$), which reduces to the ratio $A = C_{predicted}/C_{measured}$. The more A is close to 1, the better the method is in estimating cell counts. We thus built the linear model using a triplicate of growth curves for which we have both cell counts and absorbances, and then measured the accuracy as the mean A derived from all GI data within the exponential phase for three growth curves, different from those used to build the model. By repeating this procedure for 20 different combinations of growth curves, we found that on average A = (1.07 +/- 0.02) for K562 cells, in agreement with the accuracy of microsphere dilution method investigated for bacterial cells.

This result suggests that the accuracy of our method in estimating cell counts from absorbance measurements is equivalent to estimating cell counts from OD in bacteria culture.

To corroborate the robustness of our method, we tested the variability of A by changing the size of N, i.e., the number of curves used for constructing the calibration curve. Specifically, we repeated the above procedures by randomly choosing 2, 3, 5, 10 and 17 curves for the model and repeated the procedure 100 times per each condition. Figure S14 and Table S6 (below) show that increasing the number of curves used for the model helps in decreasing the dispersion of the accuracy values. However, when considering the coefficient of variation (CV) this is of the order of 0.1, varying from 0.13 (model with 2 curves) to 0.08 (model with 17 curves), suggesting that the large variability in CF and accuracy is intrinsic in the experimental working condition.

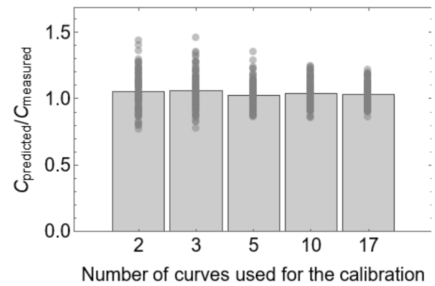

Figure S14. Comparison of the mean accuracies obtained by constructing the linear model with N = 2, 3, 5, 10, 17 curves. Dots are the average of A = *C*predicted/*C*measured from 3 curves

randomly chosen from the pool of curves not used for the model. For each condition, 100 replicates have been run, each time with *N* + 3 curves randomly chosen from the entire pool of experiments. Bars are the average of the dots. Data are from K562 cells grown in Glu at 37°C.

N curves	*A* +/- err*A*	CV
2	1.052 +/- 0.014	0.13
3	1.059 +/- 0.013	0.12
5	1.026 +/- 0.010	0.097
10	1.040 +/- 0.010	0.093
17	1.030 +/- 0.008	0.079

Table S6. Mean *A* values, standard error of the mean (err*A*) and CV (i.e. the standard deviation of *A* over its mean) obtained by constructing the linear model with different number of curves.

Overall, these results show that our method is robust in predicting the concentration of cells within the exponential phase of growth of the population independently of the number of curves used to construct the calibration curve. Moreover, the accuracies in estimating the concentration of cells are similar to predicting bacteria counts from OD.

Additional manuscript supplementary data files

Supplementary data file 1. Plots of ln(GI) and ln(*C*) over time for all biological replicates.
Supplementary data file 2. Excel file of μ_p, μ_c and CF for all biological replicates.
Supplementary software file. *Wolfram Mathematica* code used for data analysis.
Instruction on how to use the code for data analysis.
Input data script for data analysis

Supplementary References

1 Enrico Bena, C. *et al.* Initial cell density encodes proliferative potential in cancer cell populations. *Sci Rep* **11**, 6101, doi:10.1038/s41598-021-85406-z (2021).
2 Beal, J. *et al.* Robust estimation of bacterial cell count from optical density. *Commun Biol* **3**, 512, doi:10.1038/s42003-020-01127-5 (2020).
