# Supplementary material for: Mammalian cell growth characterisation by a non-invasive plate reader assay
Source: Nat Commun. 2024 Jan 2;15:57. doi: 10.1038/s41467-023-44396-4 (PMC10761699; doi:10.1038/s41467-023-44396-4)
Supplement: Supplementary file 3 — Description of Additional Supplementary Files [file 41467_2023_44396_MOESM3_ESM.pdf]

Title: Supplementary data 1
Description: Plots of ln(GI) and ln(*C*) over time for all biological replicates.

Title: Supplementary data 2
Description: Excel file of μ_p, μ_c and CF for all biological replicates.

Title: Supplementary software file.
Description: *Wolfram Mathematica* code used for data analysis.
Instruction on how to use the code for data analysis.
Input data script for data analysis
