# Supplementary material for: Mammalian cell growth characterisation by a non-invasive plate reader assay
Source: Nat Commun. 2024 Jan 2;15:57. doi: 10.1038/s41467-023-44396-4 (PMC10761699; doi:10.1038/s41467-023-44396-4)

K562 – Glu 37ºC

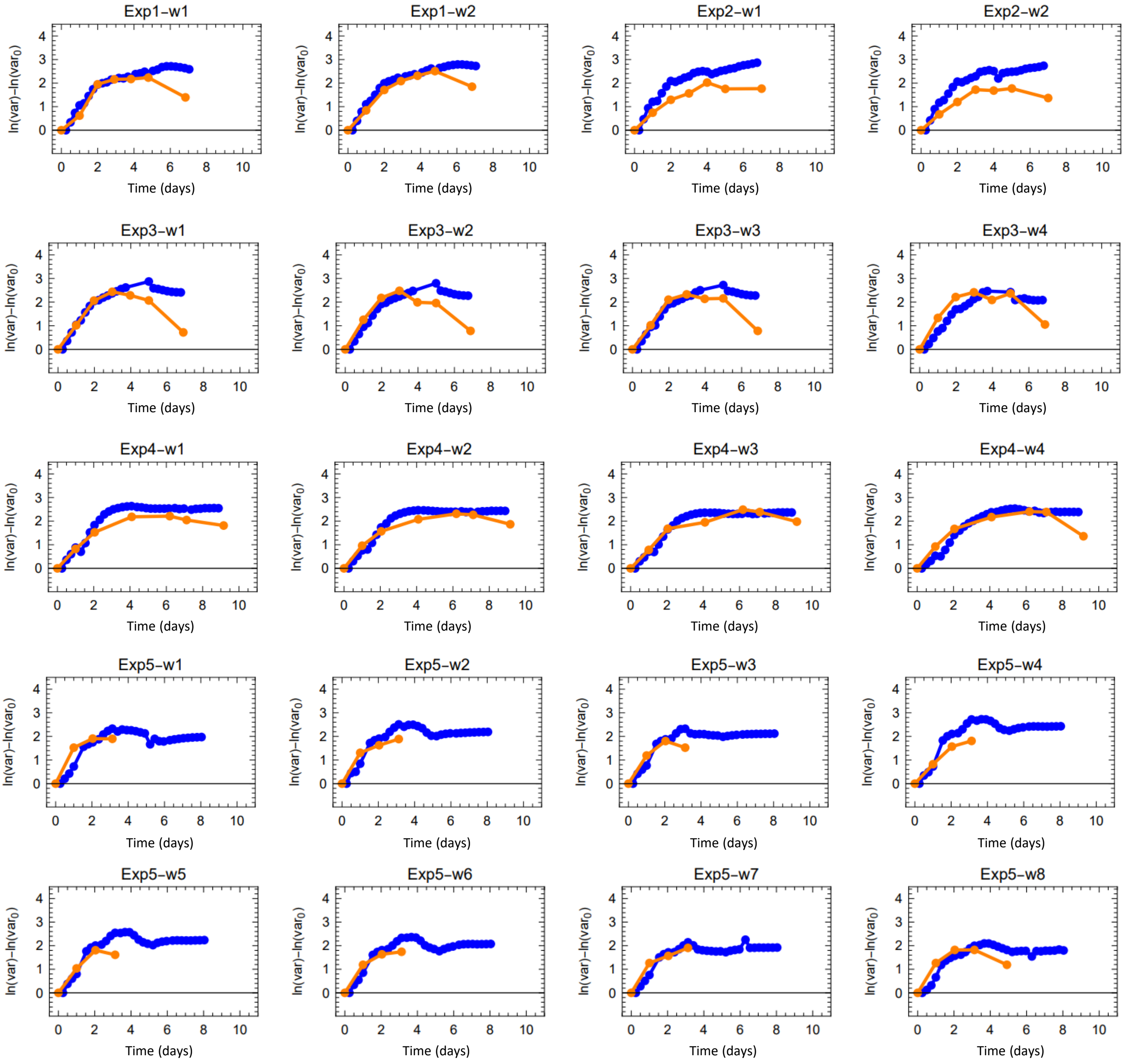

Jurkat – Glu 37ºC

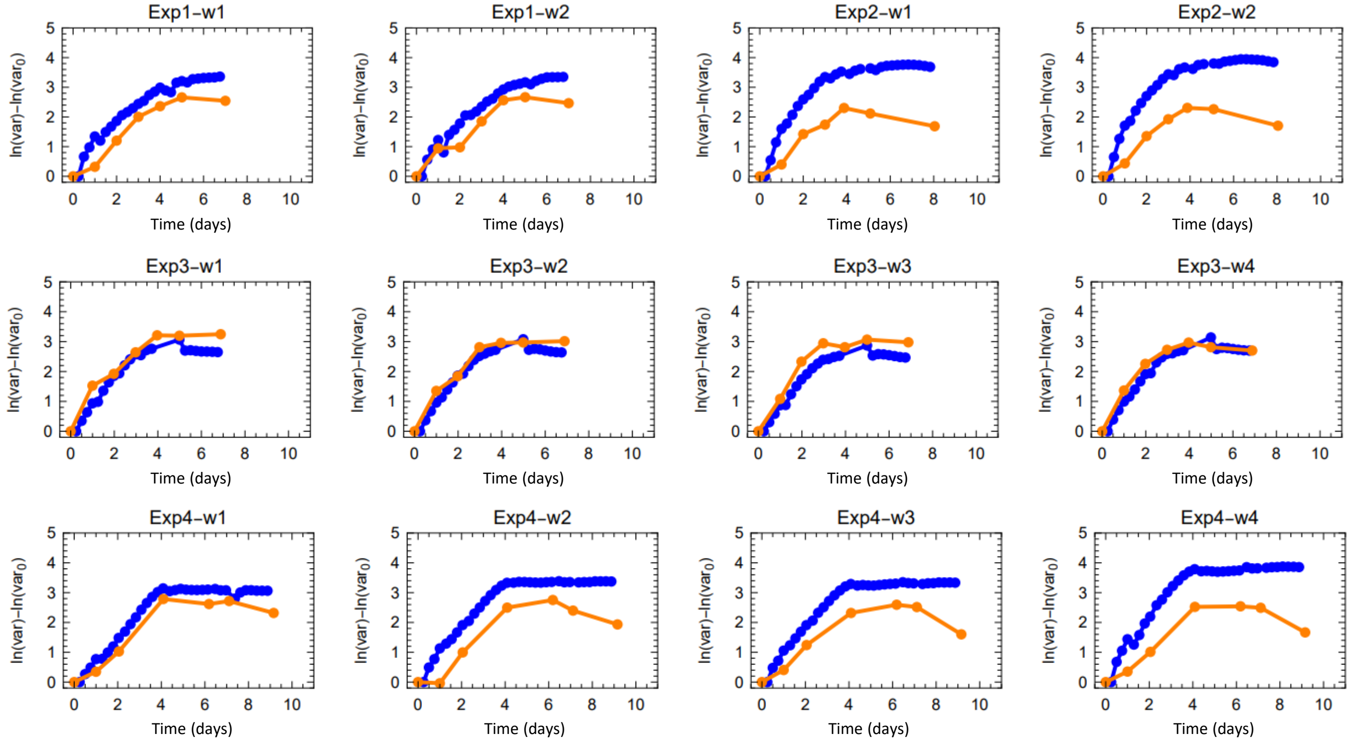

Jurkat – Glu 37°C - 0ug/ml colchicine

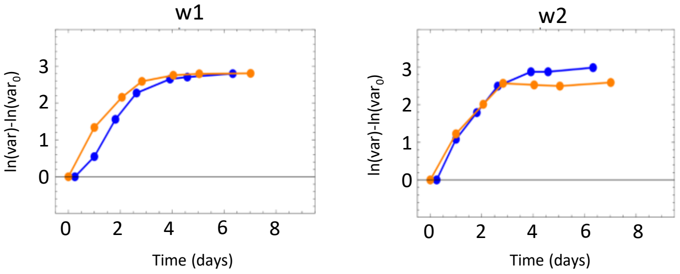

Jurkat – Glu 37°C - 0.025ug/ml colchicine

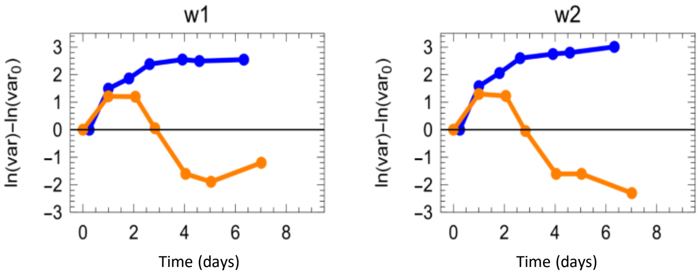

HT1080 – Glu 37°C

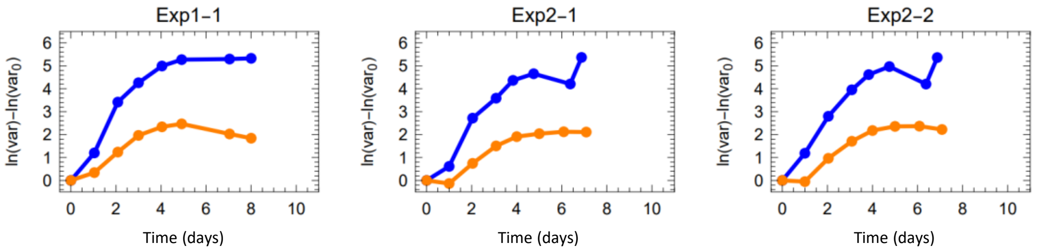

HT1080 – Glu 37°C – 15mg/L phenol red

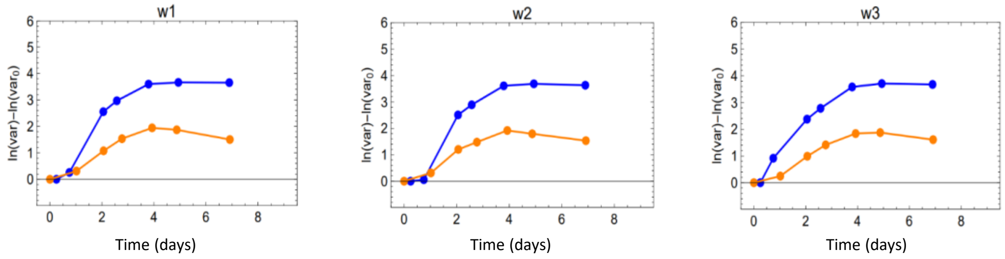

HT1080 – Glu 37°C – 5mg/L phenol red

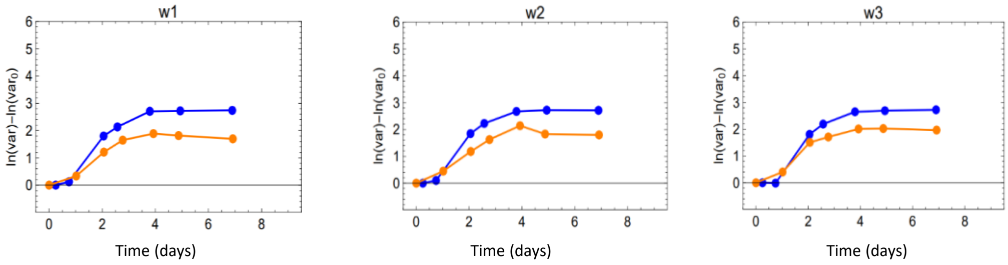

HEK293T – Glu 37ºC

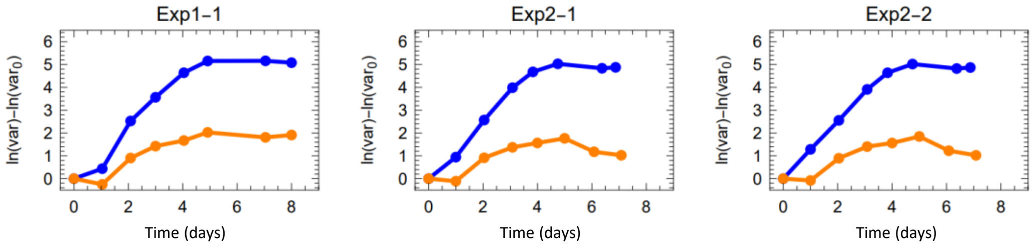

HEK293TLP-BFP – Glu 37ºC

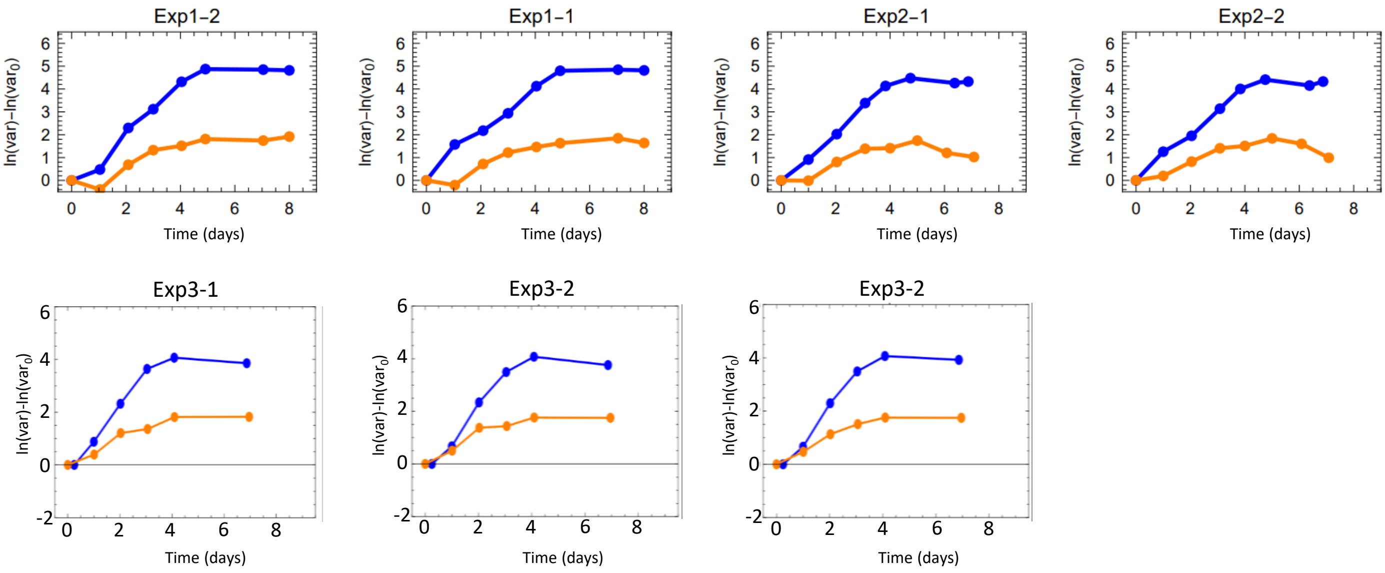

HEK293TLP-mCher – Glu 37ºC

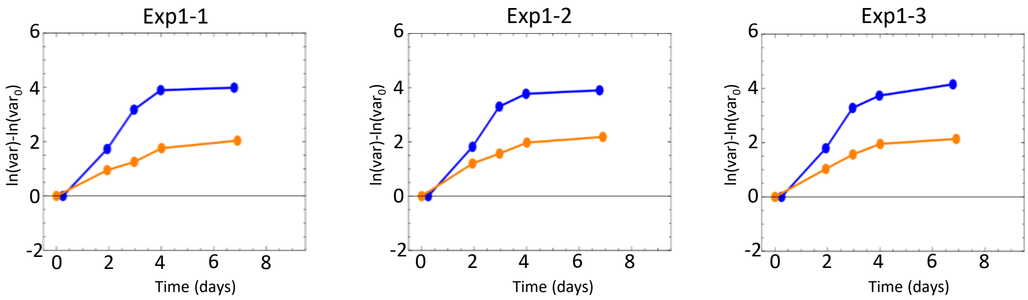

K562 – Glu 33ºC

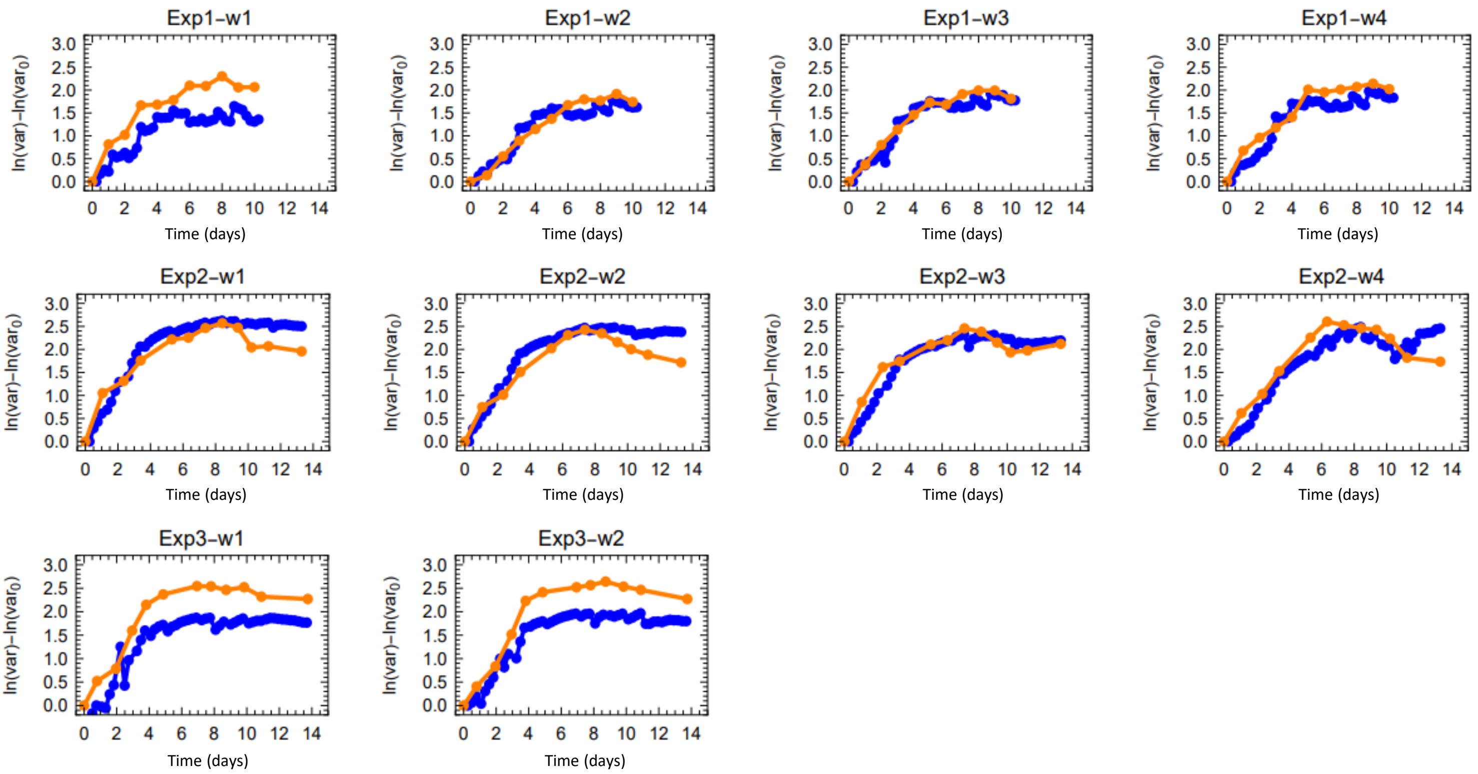

Jurkat – Glu 33ºC

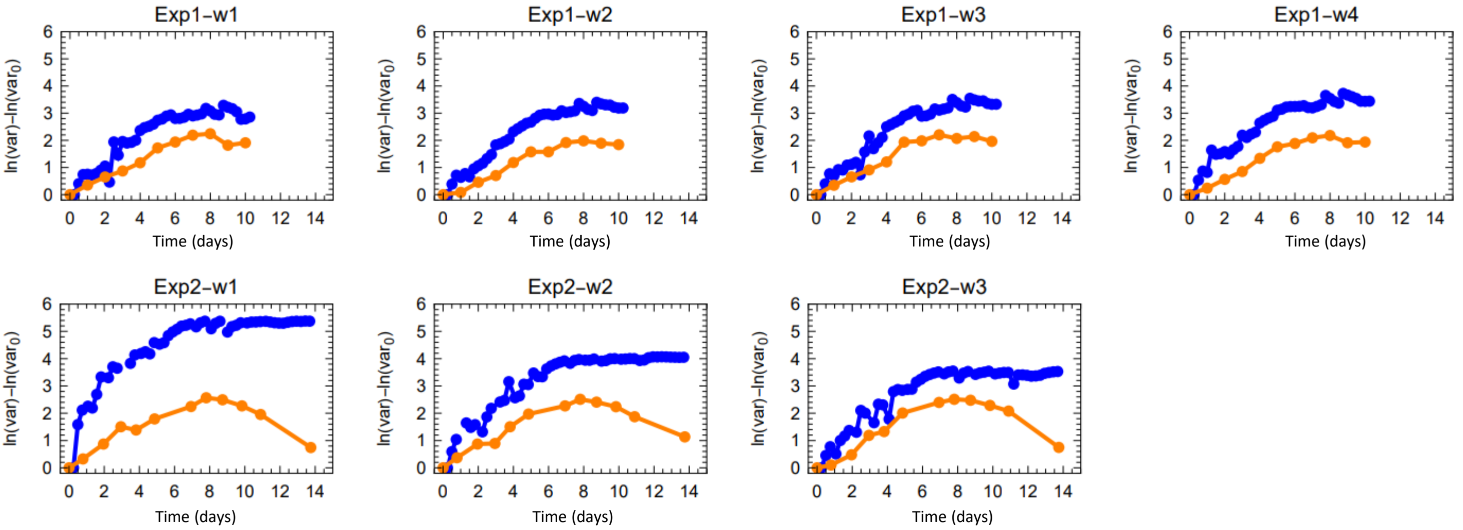

K562 – Man 37°C

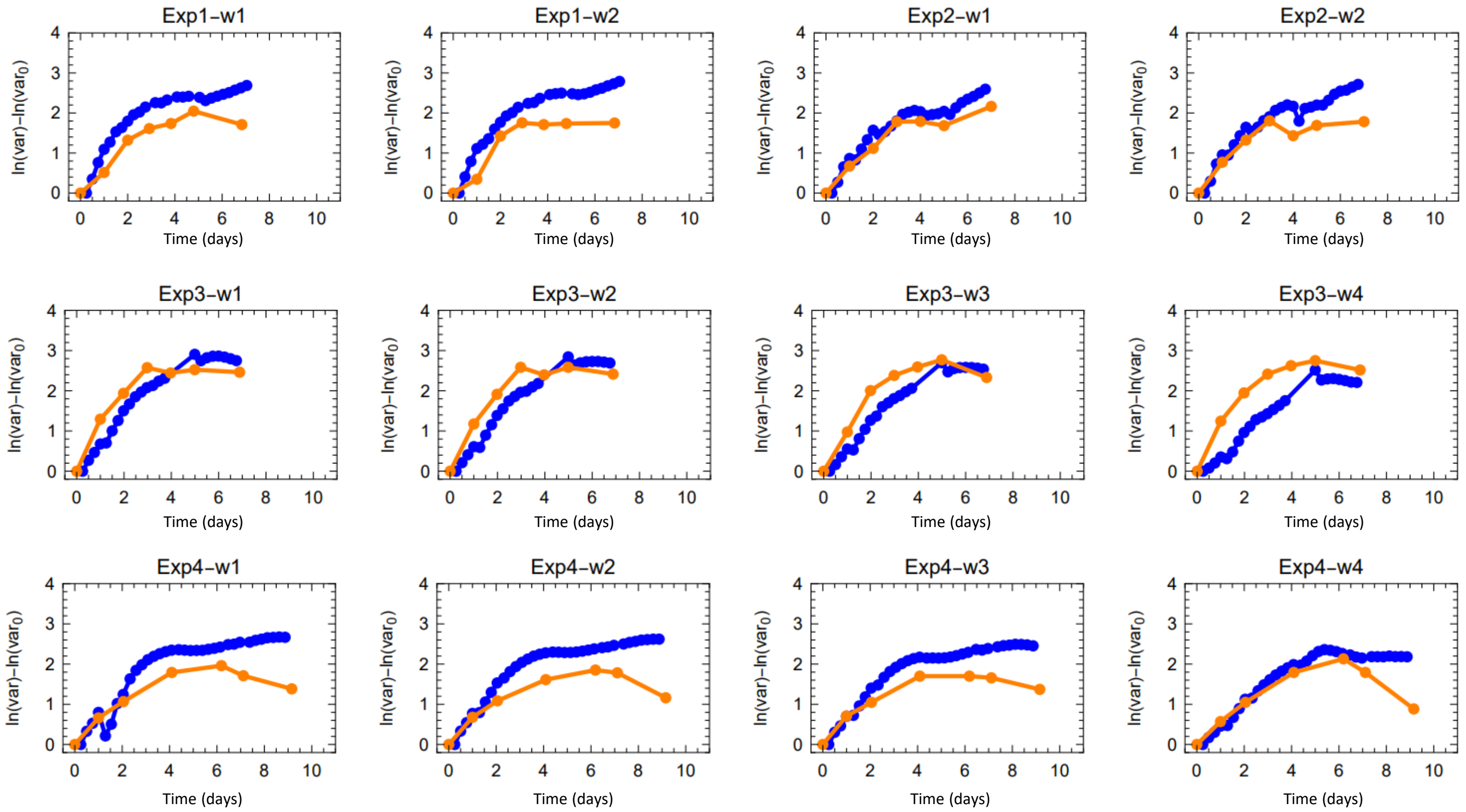

Jurkat – Man 37°C

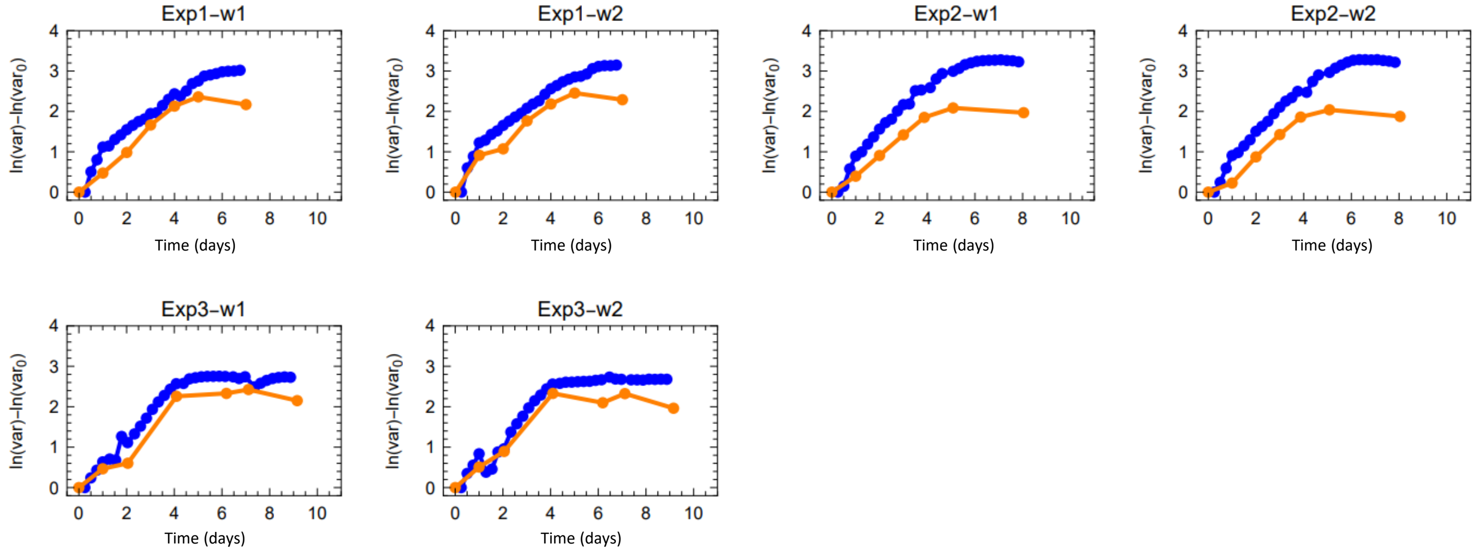

K562 – Man 33ºC

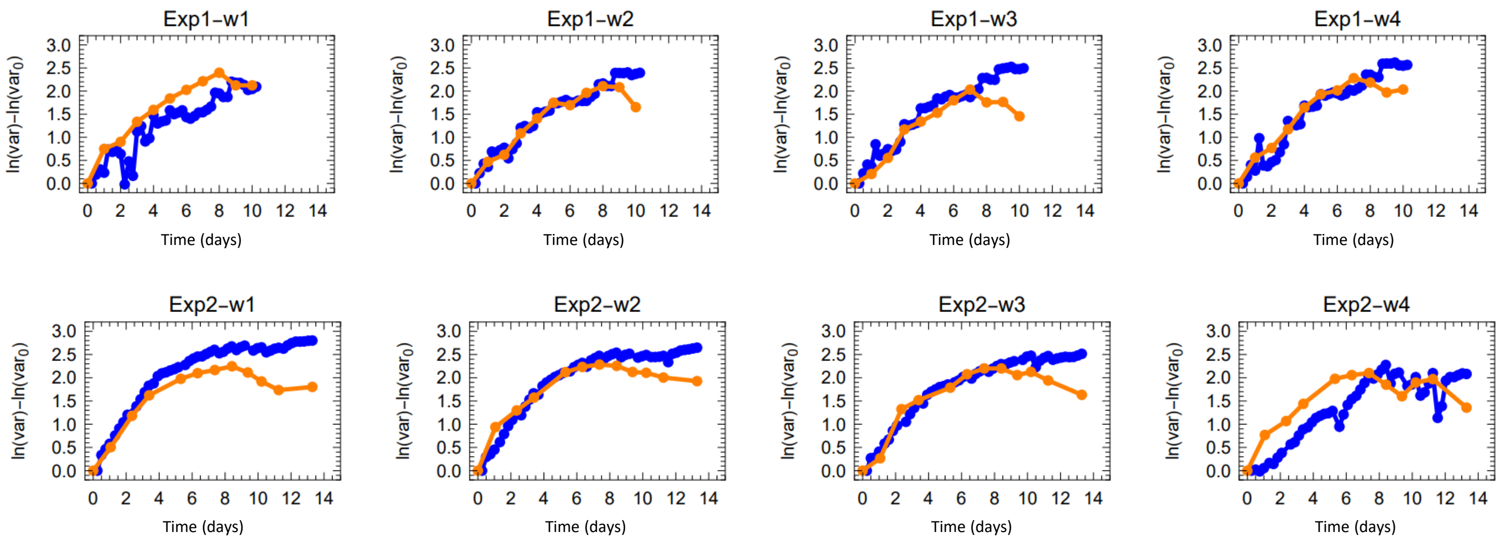

Jurkat – Man 33ºC

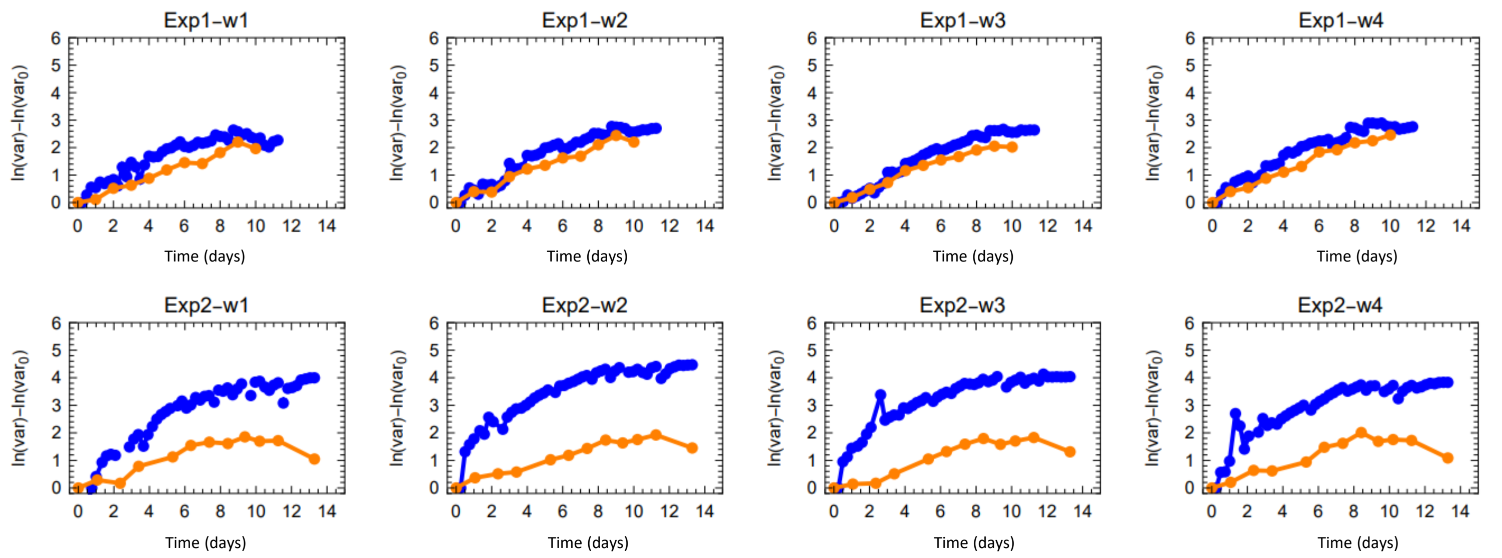

HT1080 – 0nM doxo

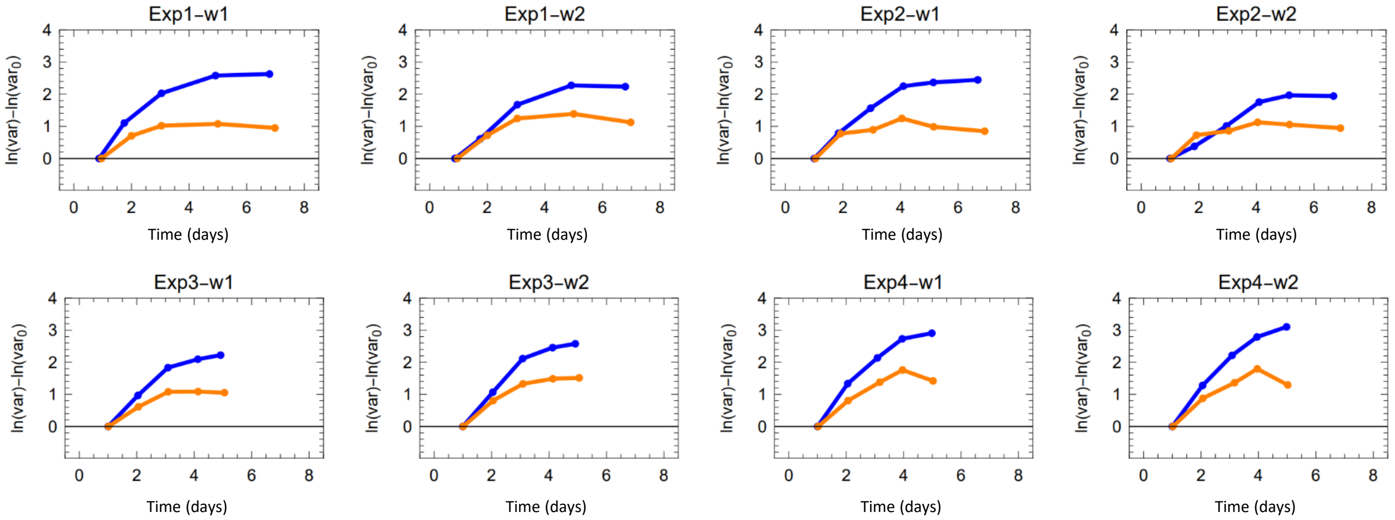

HT1080 – 10nM doxo

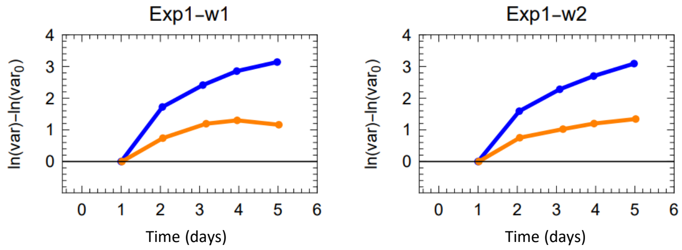

HT1080 – 25nM doxo

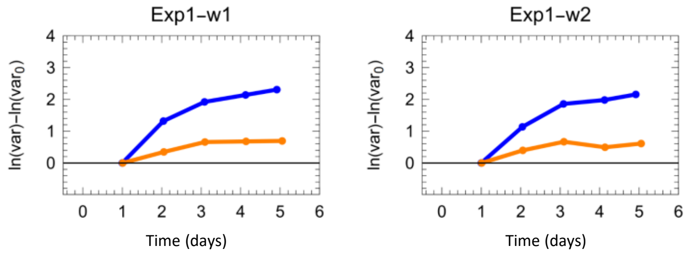

HT1080 – 50nM doxo

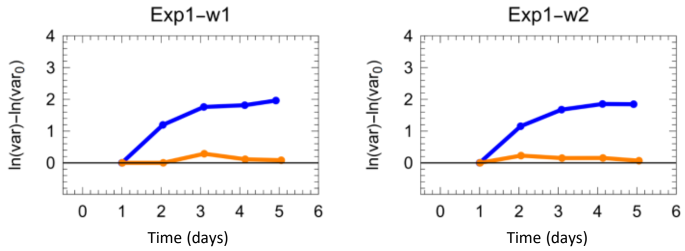

HT1080 – 75nM doxo

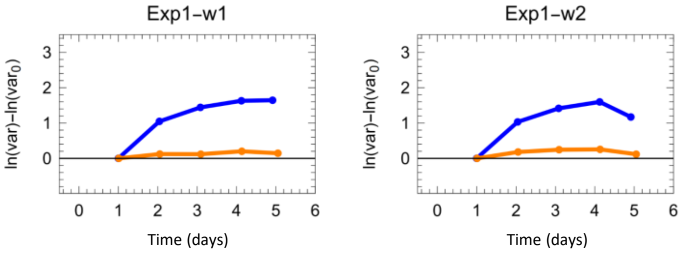

HT1080 – 100nM doxo

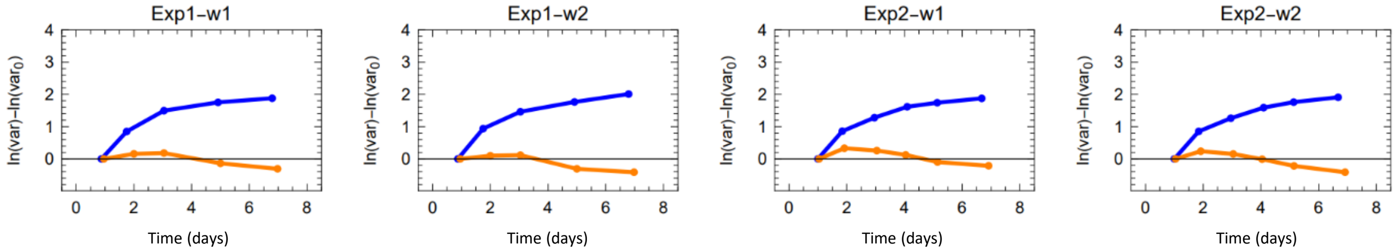

HT1080 – 500nM doxo

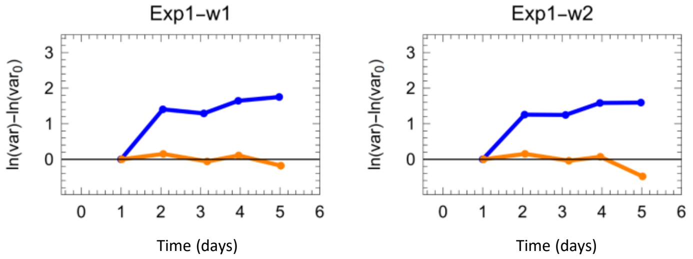

HT1080 – 750nM doxo

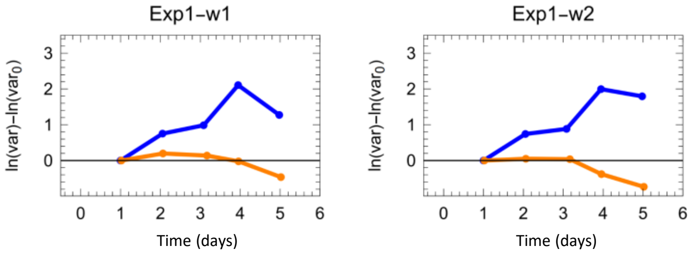

HT1080 – 1uM doxo

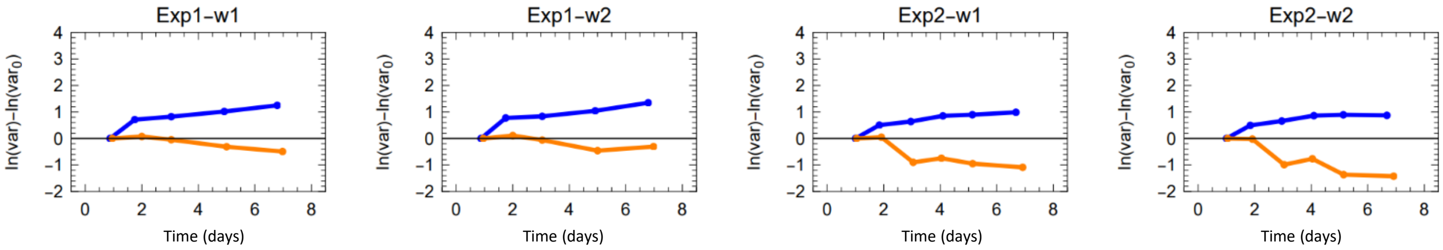

K562 – 0nM doxo

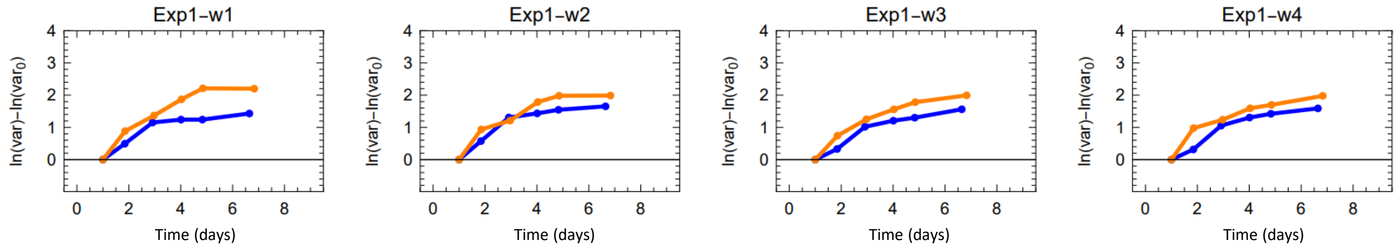

K562 – 10nM doxo

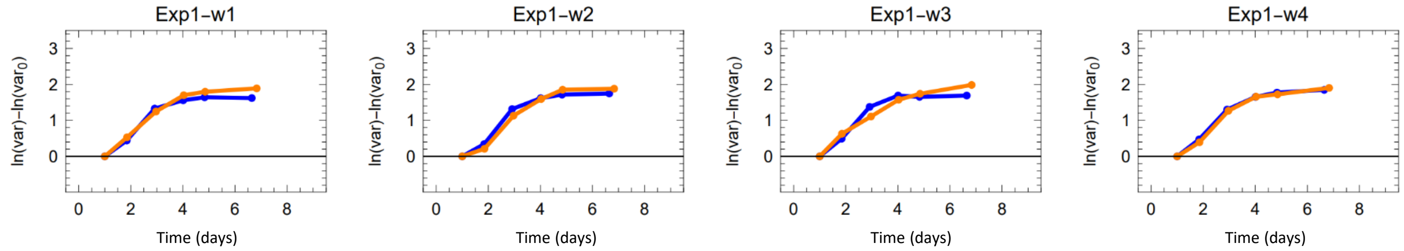

K562 – 25nM doxo

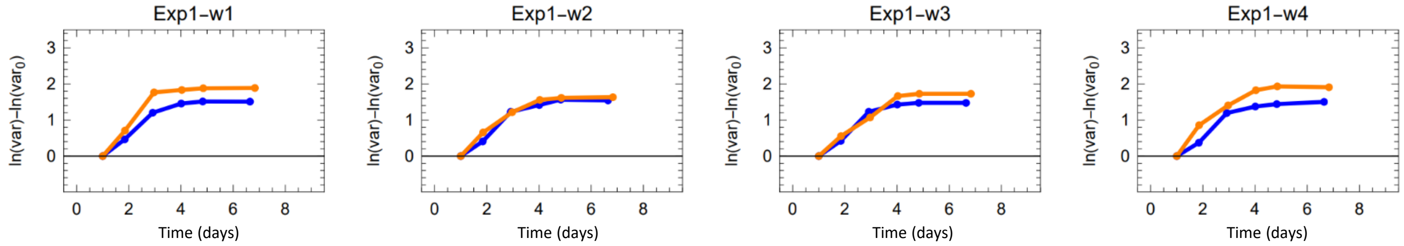

K562 – 50nM doxo

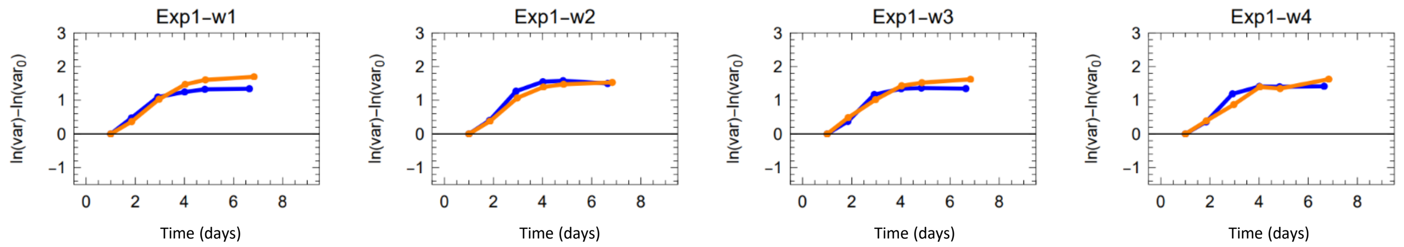

K562 – 75nM doxo

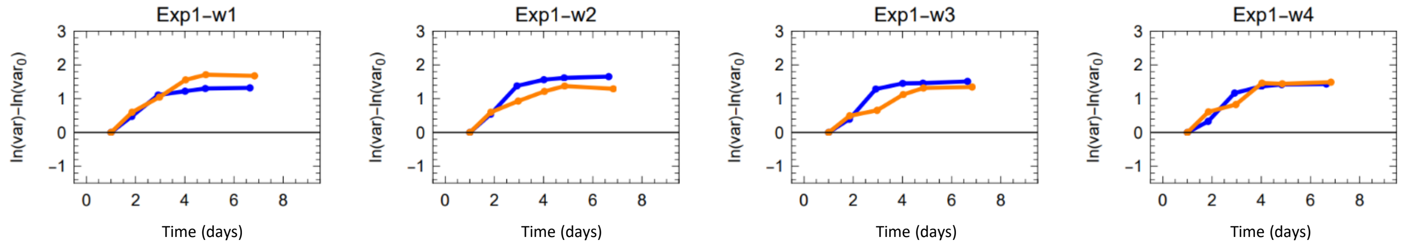

K562 – 100nM doxo

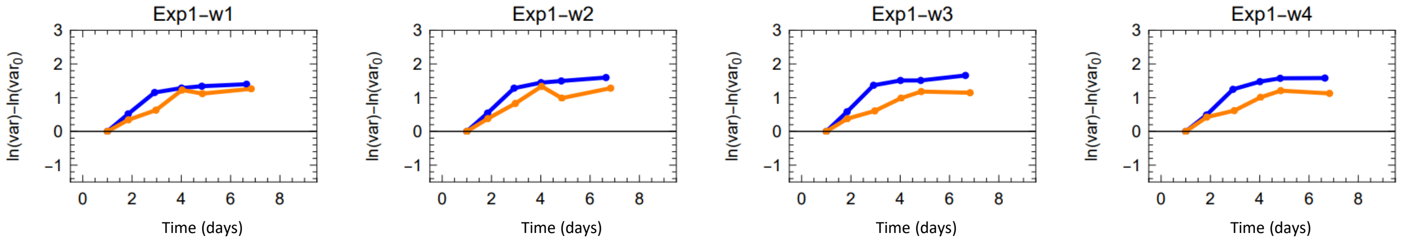

K562 – 500nM doxo

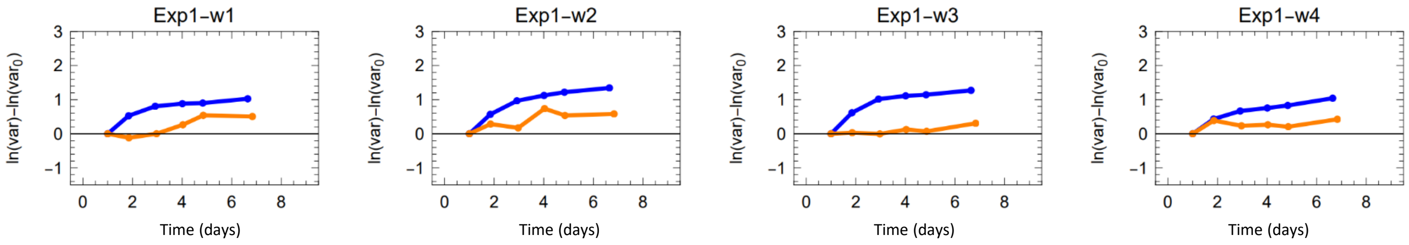

K562 – 750nM doxo

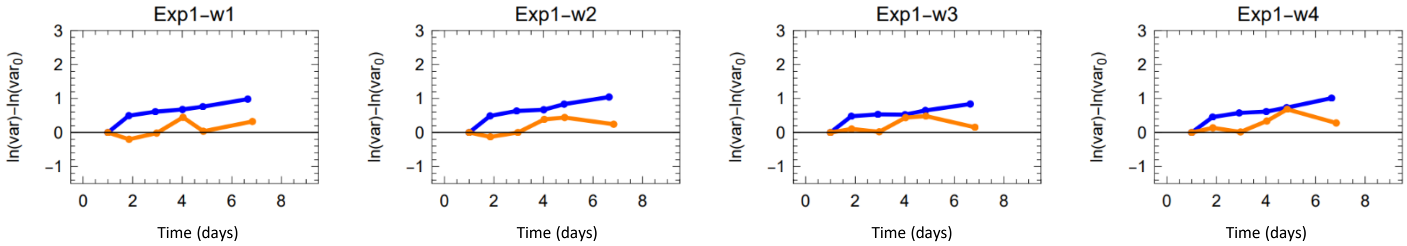

K562 – 1uM doxo

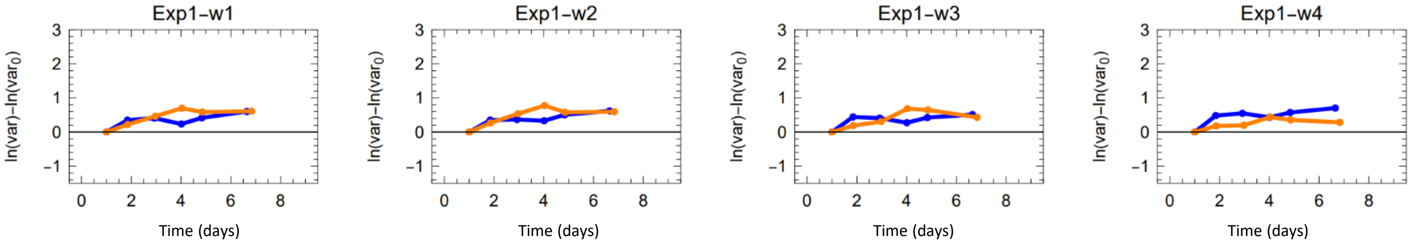

Supplement: Supplementary file 4 — Supplementary data 1 [file 41467_2023_44396_MOESM4_ESM.pdf]
