# Supplementary material for: Mammalian cell growth characterisation by a non-invasive plate reader assay
Source: Nat Commun. 2024 Jan 2;15:57. doi: 10.1038/s41467-023-44396-4 (PMC10761699; doi:10.1038/s41467-023-44396-4)
Supplement: Supplementary file 6 — Supplementary Software [file 41467_2023_44396_MOESM6_ESM.zip › Related_file_3/Code_Revision/~WRL3427.tmp]

**Mammalian cell characterisation by non-invasive plate reader assay**

Grob Alice^1,2+^, Enrico Bena Chiara^3,4+^, Di Blasi Roberto^1,2^, Pessina Daniele^1^, Matthew Sood^1^, Zhou Yunyue ^5^, Bosia Carla^3,5*^, Isalan Mark^2,6*^, Ceroni Francesca^1,2*^

1. Department of Chemical Engineering, Imperial College London, London, UK

2. Imperial College Centre for Synthetic Biology, Imperial College London, London, UK

3. Italian Institute for Genomic Medicine, Torino, Italy

4. Micalis Institute, INRA, AgroParisTech, Université Paris-Saclay, 78350 Jouy-en-Josas, France.

5. Department of Life Sciences, Imperial College London, London, United Kingdom

6. Department of Applied Science and Technology, Politecnico di Torino, Torino, Italy

+these authors contributed equally.

**Comments and guide to the use of the Notebook for data analysis**

The script is divided in the following sections:

- **FUNCTIONS**
- **IMPORT THE DATA**
- **DATA ANALYSIS (PART I - From Plate reader data to Growth curves)**
- **DATA ANALYSIS (PART II - Growth rates, Conversion Factor, relation between plate reader measurements and manual counts)**

The following notes are a guide to the use of the *Wolfram Mathematica* Notebook.

The parameters that the user will need to tune or to change are expressed in **bold**, the names of the subsections in ***Italic***.

-------------------------------------------------------------------------------------------------------------------------------

**FUNCTIONS**

This section contains the most of the functions that will be used in the script: you need to evaluate the entire section.

It is divided into smaller subsections: one to select the data from an Excel file, one to plot the data and a third one to analyse the imported data.

Other functions are defined within the script in dedicated sections.

-------------------------------------------------------------------------------------------------------------------------------

**IMPORT THE DATA**

**Important note on data formatting**

The script requires input data formatted in a specific way: in particular, it requires an Excel file in which:

- Each Sheet corresponds to a condition to study (for instance, if several sugars are present, then one sheet per sugar).
- If data of both plate reader and manual counts are present, then the last Excel Sheet must be of Manual counts and the Sheet before the last must contain measurements of the Background (i.e. measurements of wells not containing cells).
- If no manual counts data, then the last Sheet will contain the Background values.

In “IMPORT THE DATA” section the path of the directory containing the data must be written in **pathData**. By default, the Notebook takes in input the data that are in the folder *Input_Script* that is in the same directory of the notebook itself. So either move your data into this folder or change the path in **pathData**.

-------------------------------------------------------------------------------------------------------------------------------

**DATA ANALYSIS (PART I) - From Plate reader data to Growth curves**

This section is divided in several sub-sections that: analyse the Excel file, export the data for the single Excel sheets, plot and export the growth curves.

***Create directory to export data and plots***

This section automatically creates a directory in the same location of the notebook that is called “Output_Script” and contains two subfolders “Plots” and “Data”: here the plots and the data obtained with the present script will be exported.

***Select the data to analyse***

**ManualYorN** set it equal to 1 if there is the Sheet with Manual counts values, set it equal to 0 otherwise.

In the Excel file given as an example there are 2 conditions, Background and Manual counts, therefore there are

- 4 Excel Sheets imported and called: “file1”, “file2”, “fileBackg”, “fileManual”

And then

- The length of these files is then computed in the subsequent line.

As shown in the screenshot here below (relative to the condition in which also Manual counts are present):


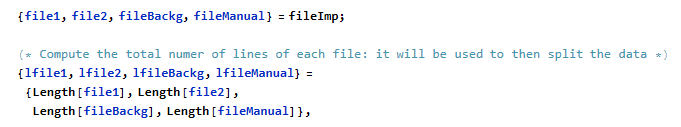


These are the files that will then be analysed.

IMPORTANT: **modify these two lists depending on how many Excel Sheets are present.** For instance, if four conditions are present (a part from Background and Manual counts), then add other names in the lists, as in the example below:


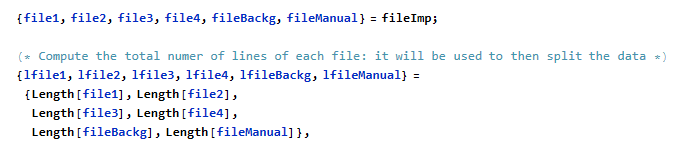


Analogously, do the same thing in case Manual counts are not present. In this case modify the subsequent lines:


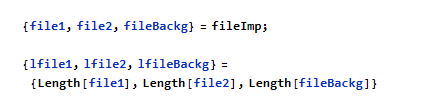


In the following, an example as the one above:


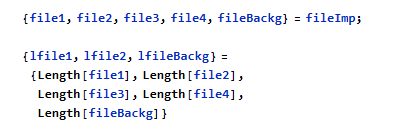


Then, concerning Plate Reader data (i.e. file1, file2, etc..) the script extracts only the lines containing the data and discards the rest. For doing this, the following parameters must be changed according to the Excel file imported:


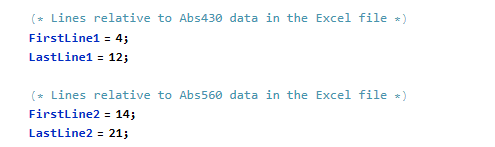


**FirstLine1**, **LastLine1** represent the lines in the Excel file relative to the begin and the end of Abs430 data.

**FirstLine2**, **LastLine2** represent the lines in the Excel file relative to the begin and the end of Abs560 data.

Change the values (actually set to 4, 12; 14, 21) according to the Excel File imported.

Analogously for the Background in the following lines:


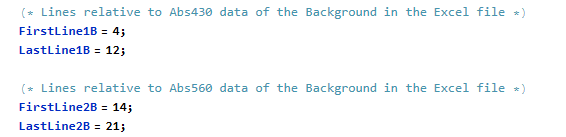


Modify **FirstLine1B**, **LastLine1B**, **FirstLine2B**, **LastLine2B** according to the imported file.

***Analyse the data and save variables of one single Excel Sheet***

From here on, only one single condition is considered. If the other conditions are needed, copy and paste all the following sections and run on another file, by modifying the variable **filetoanalyse**.

This section saves the variables of one single condition into variables for further use.

***Background analysis: compute the background value by averaging different wells***

This section computes the average background that will be then subtracted to the measurements.

It first plots the absorbance values of all wells and then gives the possibility to choose which wells to keep for computing the background (in case some wells dried out and need to be discarded). To do this, modify the variable **wellsok** by writing in the list the number of wells to analyse (numbers correspond to their order of appearance in the Excel file).

***Plot of the Abs curves of measured wells***

Plots the separated absorbances (430 and 560) of all measured wells.

***Growth curves of single wells***

This section plots the growth curves of single wells expressed as GI as a function of time after background subtraction.

The function used to plot is $functionPlot$ that can be customized by changing the following values:


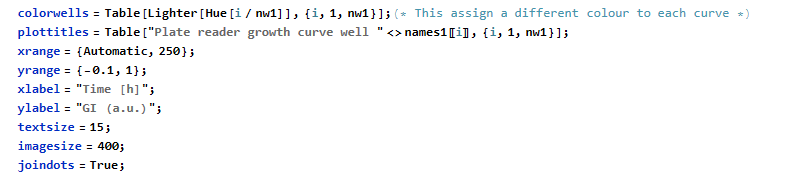


The last parameter allows to join or not the dots in the scatter plot.

***Export plots***

The following section allows to export the plots of the growth curves in the folder Plots created at the beginning of the Notebook.

Modify the following variables:

**createfolder** : set it to 1 if you want to automatically create a subfolder “DataGrowthCurves” inside “Data” folder, to save the data. Set it to 0 if the folder already exists.

**line0** = starting line to read manual counts data.

This script will export all the conditions of the manual counts while only the analysed one for Plate reader.

The growth curves will be named with the value of **condition** variable and the name of the well, as for the plots.

**exportit** value: set it to 1 if you want to export the data; to 0 otherwise.

It will automatically export Plate reader data and manual counts data into “.dat” files that will have a suffix “P” (plate reader) and “C” (manual counts).

-------------------------------------------------------------------------------------------------------------------------------

**DATA ANALYSIS (PART II)
- Growth rates, calibration (plate reader and manual counts relation), Conversion Factor**

This section allows to do the rest of the analysis, i.e. to compute growth rates, the conversion factors and the relation between manual counts and plate reader data.

It imports the data previously saved in the folder *DataGrowthCurves* and then:

***SECTION II.I: growth curves in linear and log scale***

Processes and plots plate reader growth curves in linear scale (GI normalized to the first value as a function of time) and log scale (ln(GI) normalized to its first value as a function of time) and the same for manual counts.

***SECTION II.II: relation between GI and C***

Procedure to plot and find the relation between GI and C.

*Data*

The data must be checked by the user:

**Manual counts** (the data saved include different conditions, so here you need to select the wells corresponding to the condition to compare with plate reader data).

Change the following values according to the data you wish to analyse:


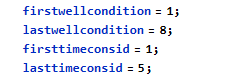


Same for Plate reader data.

*Growth curves semi log-scale GI and C together*

In this section ln(GI)-ln(G0) as a function of time and ln(C)- ln(C0) as a function of time are plotted together.

Both all Plate Reader data and only time-points corresponding to manual counts are plotted.

The time is expressed in days.

**titlesplots**: modify it by writing one name per each plot: it will be the title of the plots.

Plots and the legend can be automatically saved in the already created folder named Plots.

**exportit** value: set it to 1 if you want to export the plots; to 0 otherwise.

**namesfilestoexp**: names of the plots that you wish to export:

- The first one is the legend
- the second one the plot with all Plate Reader’s time
- the third one the plot with Plate Reader’s time points that correspond to manual counts.

**filetoexp**: the names of the variables to export.

*Compute: µC, µP, CF and linear fit of ln(GI) vs ln(C)*

The section is divided in 4 further subsection.

The first subsection (*Functions for fitting, obtaining µC, µP nad the linear relation between ln(GI) vs ln(C))* contains the functions that will be used in the following subsections to:

- select the region of fastest growth in ln(C/C0) growth curves and compute the µC (function called $\mathbf{FindBestFitExpPhase}$ )
- Select the data in ln(GI/GI0) growth curves that correspond to the region of fastest growth in ln(C/C0) (function called $\mathbf{selectdataExpPhaseGIfromC}$ ).

The second subsection ( *Select data* ) one can eventually delete outlier curves.

For instance, if you want to delete the third curve of your dataset, then write:


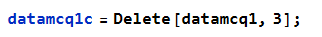


For having correspondent data in GI, the same curves deleted for C must be deleted also for GI and for the names of the wells.

If you don’t want to delete any curve, then write:


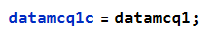


In the third subsection (*Apply functions to the data*) the data are analysed to:

1. Fit the ln(C/C0) growth curves and obtain µC.
   The only parameter that needs to be determined for this procedure is **atleast**:


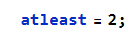

It allows to choose the minimum number of data to fit. The variable **atleast** is equal to the number of minimum data to fit – 1. For example, **atleast = 2**, means that 3 consecutive time-points are considered for the fit.

1. Select, in ln(GI/GI0) growth curves the same time-points determined in i) to obtain µP.
2. Find the linear fit between ln(GI) and ln(C ) in the region of fastest growth.
3. Plot the final best fit.

The fourth and last subsection (*Export*) is dedicated to export the plot in iv) and data of growth rates µC and µP for all the analysed wells.

*Parameters to edit in Export the plot ln(GI) vs ln(C)*:

**exportit** set it to 1 if you wish to export the data, to 0 otherwise.

**filename** = name of the file, write it between “…”

**ext** = extension of the file, for instance “.pdf” or “.jpg”.

The plot is automatically saved in the folder Plot created at the beginning of the main Section.

*Parameters to edit in Export the plot ln(GI) vs ln(C)*:

**exportit** set it to 1 if you wish to export the data, to 0 otherwise.

**Namefiletoexp** write between “…” the name of the file you with to create

**ext** extension of the file that you want to create (for instance “.csv” for a csv file, “.dat” for a dat file, etc).

The output file is automatically saved in the folder DataConversionFactors created at the beginning of the main Section.

Each line of the output file will correspond to one analysed well that will have the same name as the imported file.

***SECTION II.III - Fit of the exponential phase with a sigmoidal-shaped function***

This section allows to: i) fit sigmoidal shaped data with a modified logistic function, ii) automatically determine the exponential phase of growth and iii) linear fitting the data within the exponential phase to obtain the growth rate within the exponential phase^1^.

The function requires the following quantities as input:

**datatoan** = the data to analyse organized as one list per well

**growthrateext** = estimate for the growth rate (the order of magnitude is enough for the function for fitting to converge).

**lagext** = extimate of the lag time

**xminfitlog** = minimum value for plotting the logistic function

**xmaxfitlog** = maximum value for plotting the logistic function

**xminfitline** = minimum value for plotting the line fitting the data within the exponential phase

**xmaxfitline** = minimum value for plotting the line fitting the data within the exponential phase.

The function runs on data of one single well at a time and the output is organized in the following quantities each of them contains as many elements as analysed wells.

The output are:

dataexpPh = list of data within the exponential pahse

plotdatafit = plot of the data to fit with the modified logistic function

functionfit = result of the fit with the modified logistic function

fitparameters = parameters of the fit

errfitparameters = errors of the parameters of the fit

plotExpWind = plot of the data (in blue) with (in gray), the logistic fit, the saturation level, the boundaries of the exponential phase and the line corresponding to the maximum growth rate (tangent to the inflection point).

linearfitExpW = best fit line of the data within the exponential phase

growthRateExp = slope of the linearfitExpW

errGrowthRateExp = error of the slope of the linearfitExpW

FitGrowthRateExp = plot similar to plotExpWind, but with the edges of the exponential phase in red and the red line being the linear fit of the data within the exponential phase (the tangent to the inflection point is not shown).

xinflection = x-coordinate of the inflection point.

expWindow = all the details of the analysis of the exponential window. It contains:

- the lower bound of the exponential phase (that corresponds to the lag)
- the upper bound of the exponential phase
- the line tangent to the inflection point
- the plot as plotExpWind but without the data
- xfl the x-coordinate of the inflection point

***ACCURACY***

This section allows to calculate the accuracy as:

- the percentage of data included within the ‘minimal’ and ‘maximal’ linear fit between ln(GI) vs ln(C)
- the ratio between C_calculated_ and C_predicted_

The function needs as input:

Idall: the position of the curves under exam in the list of all curves.

Idxcombinations: the combination of positions of growth curves that one wants to use to create the model.

dataCvst

dataPvst

**REFERENCES**

1 Enrico Bena, C. et al. Initial cell density encodes proliferative potential in cancer cell populations. Sci Rep 11, 6101, doi:10.1038/s41598-021-85406-z (2021).
