# Supplementary material for: Mammalian cell growth characterisation by a non-invasive plate reader assay
Source: Nat Commun. 2024 Jan 2;15:57. doi: 10.1038/s41467-023-44396-4 (PMC10761699; doi:10.1038/s41467-023-44396-4)
Supplement: Supplementary file 7 — Reporting Summary [file 41467_2023_44396_MOESM7_ESM.pdf]

nature portfolio

Corresponding author(s): Ceroni Francesca, Mark Isalan, Carla Bosia

Last updated by author(s): 11-12-2023

Reporting Summary

Nature Portfolio wishes to improve the reproducibility of the work that we publish. This form provides structure for consistency and transparency in reporting. For further information on Nature Portfolio policies, see our Editorial Policies and the Editorial Policy Checklist.

Statistics

For all statistical analyses, confirm that the following items are present in the figure legend, table legend, main text, or Methods section.

n/a	Confirmed	
☐	☒	The exact sample size (n) for each experimental group/condition, given as a discrete number and unit of measurement
☐	☒	A statement on whether measurements were taken from distinct samples or whether the same sample was measured repeatedly
☐	☒	The statistical test(s) used AND whether they are one- or two-sided<br>*Only common tests should be described solely by name; describe more complex techniques in the Methods section.*
☐	☒	A description of all covariates tested
☐	☒	A description of any assumptions or corrections, such as tests of normality and adjustment for multiple comparisons
☐	☒	A full description of the statistical parameters including central tendency (e.g. means) or other basic estimates (e.g. regression coefficient) AND variation (e.g. standard deviation) or associated estimates of uncertainty (e.g. confidence intervals)
☐	☒	For null hypothesis testing, the test statistic (e.g. F, t, r) with confidence intervals, effect sizes, degrees of freedom and P value noted<br>*Give P values as exact values whenever suitable.*
☒	☐	For Bayesian analysis, information on the choice of priors and Markov chain Monte Carlo settings
☒	☐	For hierarchical and complex designs, identification of the appropriate level for tests and full reporting of outcomes
☒	☐	Estimates of effect sizes (e.g. Cohen's d, Pearson's r), indicating how they were calculated

Our web collection on statistics for biologists contains articles on many of the points above.

Software and code

Policy information about availability of computer code

Data collection	Plate reader (Spark Tecan). Data were acquired using the spark control software. Full protocol is available in the methods section of the manuscript.
Data analysis	Data analysis was performed using a custom Mathematica script. Full description is provided in the related file 3 and in supplementary note 1.

For manuscripts utilizing custom algorithms or software that are central to the research but not yet described in published literature, software must be made available to editors and reviewers. We strongly encourage code deposition in a community repository (e.g. GitHub). See the Nature Portfolio guidelines for submitting code & software for further information.

Data

Policy information about availability of data

All manuscripts must include a data availability statement. This statement should provide the following information, where applicable:

- Accession codes, unique identifiers, or web links for publicly available datasets
- A description of any restrictions on data availability
- For clinical datasets or third party data, please ensure that the statement adheres to our policy

Source data used to compile figures are available in the source data file. constructs and cell lines are available upon requests. Construct maps are available within the source data folder.

Research involving human participants, their data, or biological material

Policy information about studies with human participants or human data. See also policy information about sex, gender (identity/presentation), and sexual orientation and race, ethnicity and racism.

Reporting on sex and gender	N/A
Reporting on race, ethnicity, or other socially relevant groupings	N/A
Population characteristics	N/A
Recruitment	N/A
Ethics oversight	N/A

Note that full information on the approval of the study protocol must also be provided in the manuscript.

Field-specific reporting

Please select the one below that is the best fit for your research. If you are not sure, read the appropriate sections before making your selection.

[x] Life sciences [ ] Behavioural & social sciences [ ] Ecological, evolutionary & environmental sciences

For a reference copy of the document with all sections, see nature.com/documents/nr-reporting-summary-flat.pdf

Life sciences study design

All studies must disclose on these points even when the disclosure is negative.

Sample size	Number of biological repeats and sample size are reported in Table S3. Statistical methods adopted are described in the figure captions and individual values reported.
Data exclusions	A data esclusion statement is present in the methods section: Data exclusion. Outliers with GI < GI0 or C < C0, where C0 is the initial concentration of cells and GI0 is GI normalised to zero at six hours were excluded. When cell count was performed in parallel to absorbance measurements, first GI measurements upon restarting the plate reader were discarded. GI growth curves with negative value for GI at six hours were excluded from the analysis.
Replication	Number of biological repeats and sample size are reported in Table S3
Randomization	N/A
Blinding	N/A

Reporting for specific materials, systems and methods

We require information from authors about some types of materials, experimental systems and methods used in many studies. Here, indicate whether each material, system or method listed is relevant to your study. If you are not sure if a list item applies to your research, read the appropriate section before selecting a response.

Materials & experimental systems

n/a	Involved in the study	
[x]	[ ]	Antibodies
[ ]	[x]	Eukaryotic cell lines
[x]	[ ]	Palaeontology and archaeology
[x]	[ ]	Animals and other organisms
[x]	[ ]	Clinical data
[x]	[ ]	Dual use research of concern
[x]	[ ]	Plants

Methods

n/a	Involved in the study	
[x]	[ ]	ChIP-seq
[x]	[ ]	Flow cytometry
[x]	[ ]	MRI-based neuroimaging

Eukaryotic cell lines

Policy information about cell lines and Sex and Gender in Research

Cell line source(s)	Suspension cells [K562 (ATCC CCL-243) and Jurkat (ATCC TIB-152)] and adhesion HEK293T (ATCC CRL-3216) and HT1080 (ATCC CCL-121) were purchased from ATCC. HEK293TLP-EBFP (HEK 293T AAVS1 LP), were a kind gift from D.M. Fowler, HEK293TLP-mCherry (bearing genomic expression of mCherry) were generated in-house from HEK293T (see Source data for map and sequence)
Authentication	Cells were purchased from ATCC and no authenticated further
Mycoplasma contamination	HEK293T were screened for mycoplasma. The other cell lines were not tested.
Commonly misidentified lines (See ICLAC register)	N/A

Plants

Seed stocks	N/A
Novel plant genotypes	N/A
Authentication	N/A
